# Development of a 17-DMAG-Loaded Carboxymethylcellulose Gel for *In Vivo* Treatment of Cutaneous Leishmaniasis

**DOI:** 10.1021/acsomega.6c00447

**Published:** 2026-05-05

**Authors:** Kercia Pinheiro Cruz, Mariana Rolemberg Gueudeville Silveira, Igor Rolemberg Gueudeville Silveira, Jade Liz Ferreira Mendes Souza, Marina Faillace De Amorim, Alan Gualberto De Souza De Freitas De Pinho, Ana Luiza de Jesus Cordeiro, Izabella Gouveia Oliveira, Isadora dos Santos Lima, Claudia Ida Brodskyn, Juliana Perrone Bezerra de Menezes, Deborah Bittencourt Motte, Henrique Rodrigues Marcelino, Fabio Rocha Formiga, Washington Luis Conrado dos Santos, Thamires Quadros Froes, Patricia Sampaio Tavares Veras

**Affiliations:** † Laboratory of Host-Parasite Interaction and Epidemiology, Gonçalo Moniz Institute, Fiocruz-Bahia, Rua Waldemar Falcão, 121, bairro Candeal, Salvador, BA CEP 40296-710, Brazil; ‡ National Institute of Science and Technology of Tropical Diseases (INCT-DT), National Council for Scientific Research and Development (CNPq), Rua Augusto Viana, s/n, Hospital Universitário Professor Edgard Santos, 5° andar, Canela, Salvador, BA, CEP 40110-160, Brazil; § Department of Preventive Veterinary Medicine and Animal Production, School of Veterinary Medicine and Animal Science, Federal University of Bahia, Salvador 40170-110, Bahia, Brazil; ∥ Department of Medicines, College of Pharmacy, Federal University of Bahia, Salvador 40170-115, Bahia, Brazil; ⊥ Aggeu Magalhães Institute, Oswaldo Cruz Foundation (FIOCRUZ), Recife 50670-420, Pernambuco, Brazil; # Faculty of Medical Sciences, University of Pernambuco, Recife 50100-130, Pernambuco, Brazil; ∇ Laboratory of Structural and Molecular Pathology, Gonçalo Moniz Institute, Oswaldo Cruz Foundation (FIOCRUZ), Rua Waldemar Falcão, 121, bairro Candeal, Salvador - BA, CEP: 40296-710, Brazil; ○ Department of Pathology and Forensic Medicine, Bahia Medical School, Federal University of Bahia, Salvador 40110-906, Bahia, Brazil

## Abstract

Cutaneous leishmaniasis (CL) is a neglected tropical disease for which safer and more effective therapeutic options are urgently needed. Heat shock protein 90 (Hsp90) inhibitors have emerged as promising antileishmanial agents. Among geldanamycin derivatives, 17-dimethylaminoethylamino-17-demethoxygeldanamycin (17-DMAG) has previously demonstrated potent activity against *Leishmania braziliensis*. Here, we evaluated the therapeutic potential of a topical 17-DMAG formulation for CL. The compound exhibited low cytotoxicity toward human keratinocytes (HaCaT) and THP-1 macrophages cell-lines. A carboxymethylcellulose (CMC) hydrogel containing 17-DMAG showed physicochemical stability for up to 90 days at 4 and 25 °C, with diffusion-controlled drug release. In BALB/c mice infected with *L. braziliensis*, topical treatment induced mild and transient local inflammation, without systemic toxicity. Notably, the 0.10 mg/g formulation reduced lesion size by up to 47% and achieved 80% complete healing by week 3. These findings support topical 17-DMAG formulation as a safe, effective, and noninvasive therapeutic approach for experimental CL.

## Graphical abstract

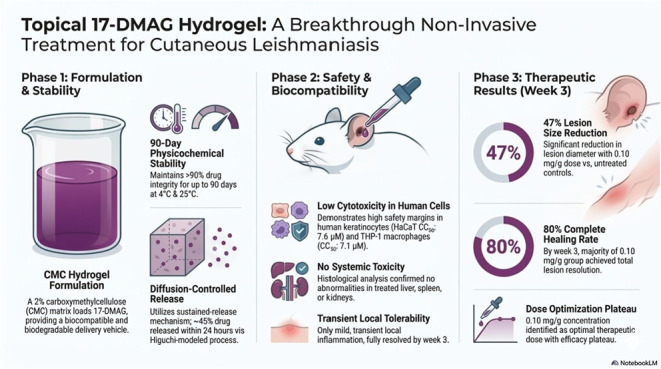

## Introduction

Leishmaniasis comprises a group of neglected tropical diseases caused by *Leishmania* spp., endemic in 97 countries, particularly in Africa, Asia, and South America.^1–3^ According to the World Health Organization (WHO), the disease is classified into visceral leishmaniasis (VL) and cutaneous leishmaniasis (CL), the latter being the most prevalent form and encompassing localized cutaneous, mucocutaneous, disseminated, and diffuse clinical presentations.^2,4–8^ Although rarely fatal, CL causes chronic skin lesions that significantly impair quality of life due to disfigurement, social stigma, and psychological consequences, contributing to morbidity and work absenteeism.^1,2,9,10^ Current treatments for CL, including pentavalent antimonials, amphotericin B, and miltefosine, are limited by severe adverse effects, invasive administration routes, and high costs, underscoring the urgent need for safer and more accessible therapeutic alternatives.^11,12^

Heat shock protein 90 (Hsp90) is a highly conserved molecular chaperone essential for the folding and stabilization of multiple client proteins, making it an attractive therapeutic target in cancer, inflammatory diseases, and parasitic infections.^13,14^ Over the past decade, ansamycin benzoquinone Hsp90 inhibitors, such as geldanamycin, 17-AAG, and 17-DMAG have consistently demonstrated leishmanicidal activity *in vitro* and *in vivo*.^15–18^ These compounds reduce *Leishmania* viability at concentrations that are nontoxic to host cells and decrease intracellular parasite burden in macrophages.^15–17^ In murine models of *Leishmania braziliensis* infection, 17-AAG treatment reduced lesion size and parasite load at the infection site, although limited effects were observed in draining lymph nodes.^16^

More recently, the water-soluble geldanamycin derivative 17-DMAG was shown to exhibit potent activity against *L. braziliensis* within bone marrow–derived macrophages at nanomolar concentrations, with low host-cell toxicity and a high selectivity index.^18^ *In vivo*, systemic administration of 17-DMAG resulted in complete lesion resolution, clearance of parasite burden in both ears and lymph nodes, and a marked reduction in pro-inflammatory cytokines (IL-6, IFN-γ, TNF), as well as IL-10.^18^ These findings support 17-DMAG as a promising antileishmanial agent with combined leishmanicidal and immunomodulatory effects.

Topical therapy has emerged as an attractive, noninvasive strategy for the treatment of localized skin diseases, including CL.^6,19,20^ According to the Pan American Health Organization, topical formulations such as creams, ointments, and gels offer advantages over systemic and other local therapies, including reduced systemic toxicity, ease of use, lower cost, and the potential for self-administration.^21–24^ These benefits may reduce hospital visits and treatment abandonment, particularly in resource-limited settings. In line with this perspective, both the WHO and the Drugs for Neglected Diseases initiative (DNDi) recommend topical treatments as first-line options for uncomplicated CL, reserving systemic therapy for refractory or severe cases.^11,25^

Gels are three-dimensional polymeric networks capable of sustained drug release and prolonged skin residence, while also promoting moisture retention and potentially reducing scar formation.^26,27^ Leveraging these properties, and considering the favorable pharmacokinetic profile of 17-DMAG compared with other geldanamycin derivatives,^13,28,29^ we hypothesized that a carboxymethylcellulose (CMC) gel containing 17-DMAG could serve as an effective topical therapy for CL. In this study, we evaluated the *in vivo* toxicity and therapeutic efficacy of a semisolid 17-DMAG–loaded CMC gel applied topically in a BALB/c murine model of *L. braziliensis*infection.

## Results

### *In Vitro* Cytotoxicity

17-DMAG reduced viability of THP-1 and HaCaT cells in a concentration-dependent manner (Alamar Blue), with comparable CC_50_ values of 7.1 ± 0.2 μM (THP-1) and 7.6 ± 0.2 μM (HaCaT) (Figure 1).

**Figure 1:**
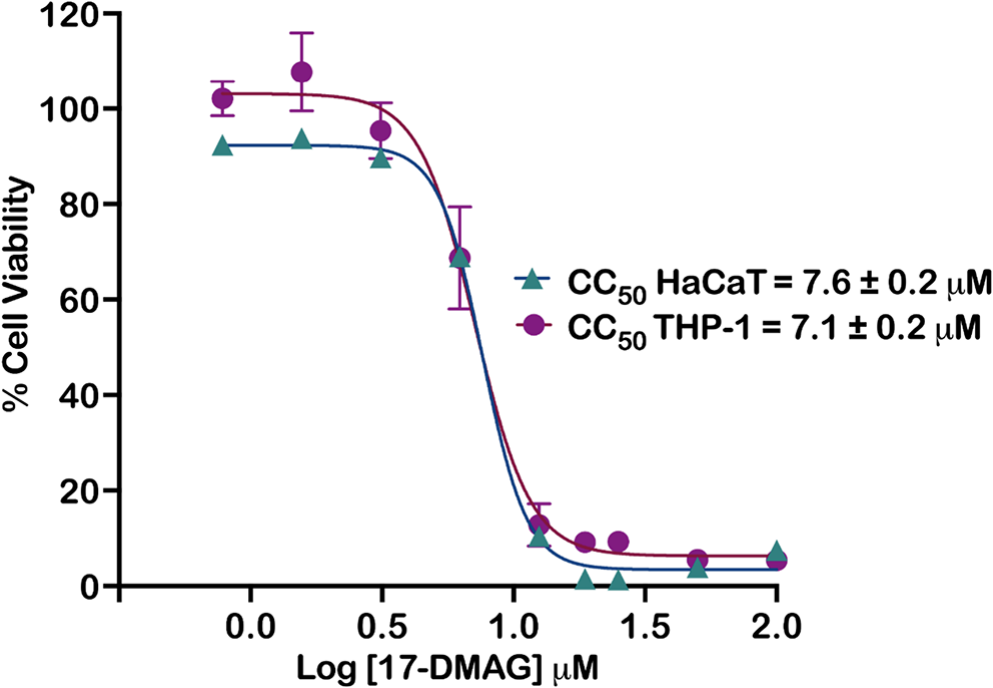
Cytotoxicity of 17-DMAG against human cell lines. THP-1 cells (5 × 10^4^ cells/well) and HaCaT cells (3 × 10^4^ cells/well) were seeded in 96-well plates and exposed to concentrations of 17-DMAG that varied from 100 to 0.78 μM. After 48 h, 20 μL of Alamar Blue reagent was added, and the cells were incubated for an additional 24 h. Reduction of Alamar Blue was measured at the wavelengths of 570 and 600 nm using a SPECTRAmax-340PC spectrophotometer. Data represent median values with 95% confidence intervals from three independent experiments performed in triplicate. CC_50_ values were calculated by nonlinear regression using a four-parameter model (variable slope) applied to normalized response data, as available in GraphPad Prism software v10.4.0 (GraphPad Software, San Diego, CA, USA).

### Characterization and Stability of 17-DMAG–Loaded CMC Gels

The 17-DMAG-loaded CMC gels were successfully prepared, showing a purple color with intensity proportional to the drug concentration. Macroscopic characterization revealed that samples stored at 4 and 25 °C retained their chemical and physical characteristics, whereas storage at 37 °C resulted in a noticeable loss of color and a marked reduction in viscosity, indicative of polymer or drug degradation (Figure 3S-A).

Quantitative stability testing showed that formulations stored at 4 and 25 °C remained within ± 10% of initial drug content over 90 days across all tested loads (0.15–0.30 mg/g) (Figure 2A–B). Cold storage provided the highest retention (consistently >93%). At 25 °C, retention remained high for 0.15 and 0.25 mg/g (>99%), while the 0.20 mg/g formulation showed moderate loss (86.4%). In contrast, storage at 37 °C caused marked degradation across all concentrations, with only 44.2 ± 0.1%, 37.8 ± 0.0%, 41.5 ± 0.0%, and 41.1 ± 0.2% remaining after 90 days for 0.15, 0.20, 0.25, and 0.30 mg/g, respectively (Figures 2C and 3S–B).

**Figure 2:**
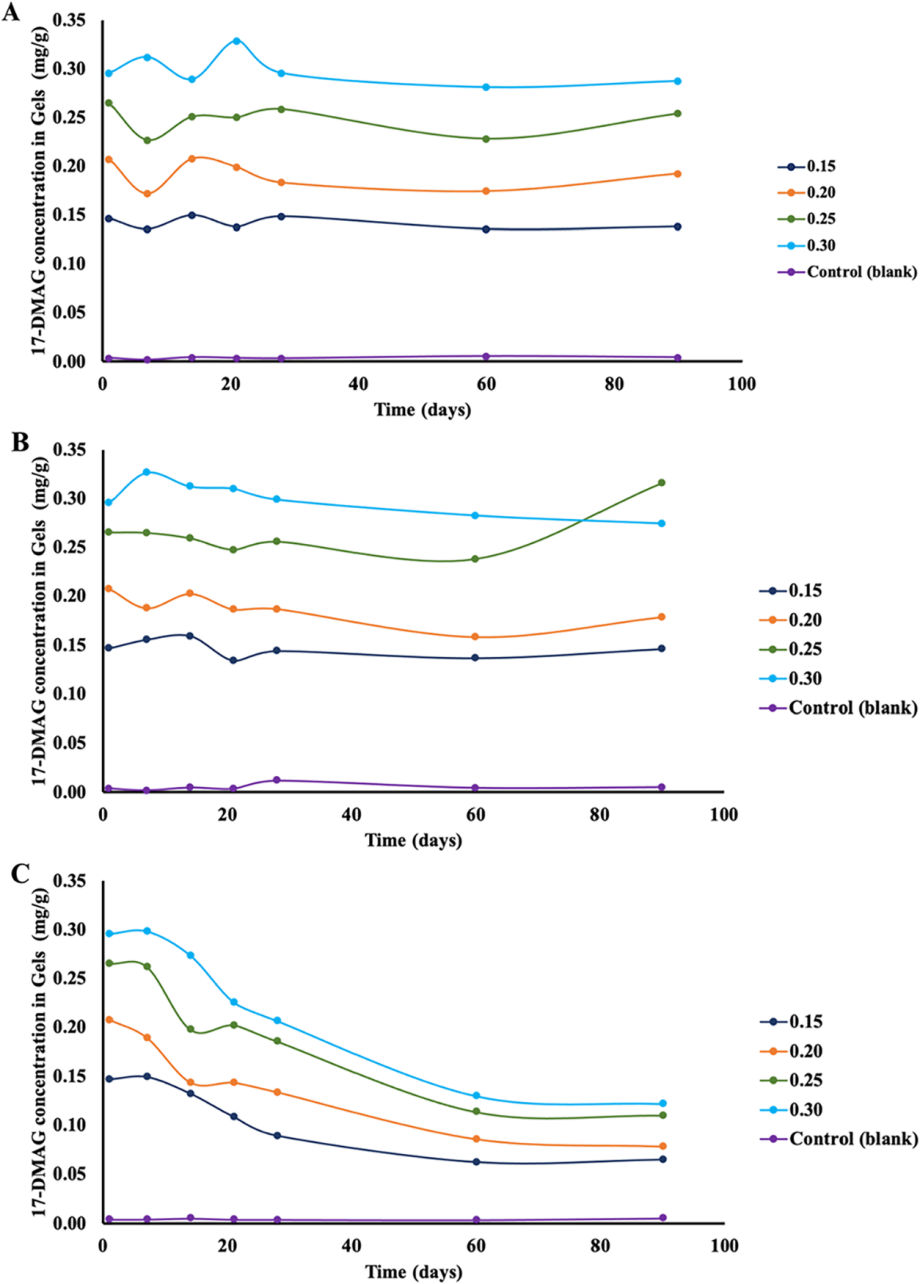
Stability of 17-DMAG in CMC hydrogels under different storage conditions. 17-DMAG was incorporated into hydrogels at 0.15 (dark blue), 0.20 (orange), 0.25 (green), and 0.30 mg/g (light blue) by dilution in distilled water, followed by addition of 2% carboxymethylcellulose (CMC). A drug-free hydrogel (CMC + distilled water) served as the control (orange). Drug stability was assessed on days 1, 7, 14, 21, 28, 60, and 90 using spectrophotometric analysis (SPECTRAmax-340PC, λ = 340 nm). Each point represents the mean of a single experiment performed in triplicate. (A) 17-DMAG stability at 4 °C; (B) 17-DMAG stability at 25 °C; and (C) 17-DMAG stability at 37 °C.

### *In Vitro* Release from the CMC Gel

For the *in vitro* release from the CMC gel, the 0.33 mg/g concentration of 17-DMAG was selected to maintain drug levels above the limit of quantification (Figure 2S). During the first 2 h of the drug release experiment, all measured values were above the detection limit of the analytical method but below its quantification limit (Figure 3). Quantifiable data showed that ∼45% of 17-DMAG diffused across the membrane between 4 and 24 h, with a diffusion-controlled profile well described by the Higuchi model (Mt = 11.947√*t* – 14.21; adjusted *R* ^2^ = 0.9971; *p* = 0.0009) (Figure 2), consistent with sustained release.

**Figure 3:**
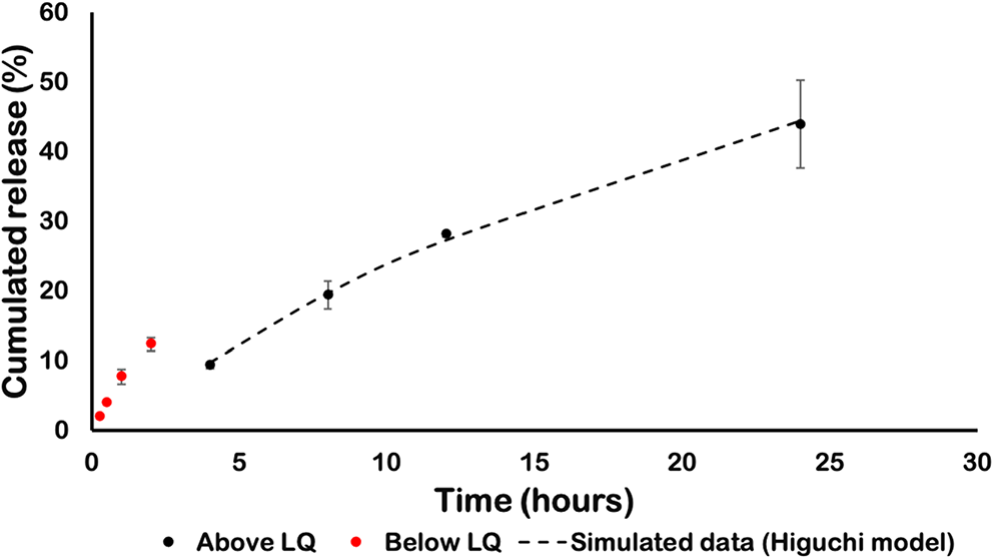
Kinetics of 17-DMAG release from the CMC gel. 17-DMAG was incorporated into gel at 0.33 mg/g. The black points refer to concentrations in the linearity range. The red points are below the limit of quantification. The traced line corresponds to the simulated data based on the modeling through the Higuchi model.

### *In Vivo* Local Tolerability

Vehicle-treated mice (control-blank) showed minimal change in ear thickness throughout follow-up (week 4:0.057 ± 0.030 mm), indicating good tolerability of the formulation base (Figure 4A). In contrast, topical 17-DMAG produced a dose-dependent, although discrete, increase in ear thickness, with peak responses at week 2. The 0.05 mg/g dose remained similar to vehicle (week 4:0.09 ± 0.01 mm). Intermediate doses showed transient increases peaking at week 2 (0.10 mg/g: 0.17 ± 0.02 mm; 0.15 mg/g: 0.24 ± 0.03 mm) that largely resolved by week 4 (0.10 mg/g: 0.14 ± 0.09 mm; 0.15 mg/g: 0.10 ± 0.04 mm). The highest dose (0.20 mg/g) showed the largest peak (week 2:0.367 ± 0.033 mm) and remained elevated at week 4 (0.19 ± 0.06 mm) (Figure 4A).

**Figure 4:**
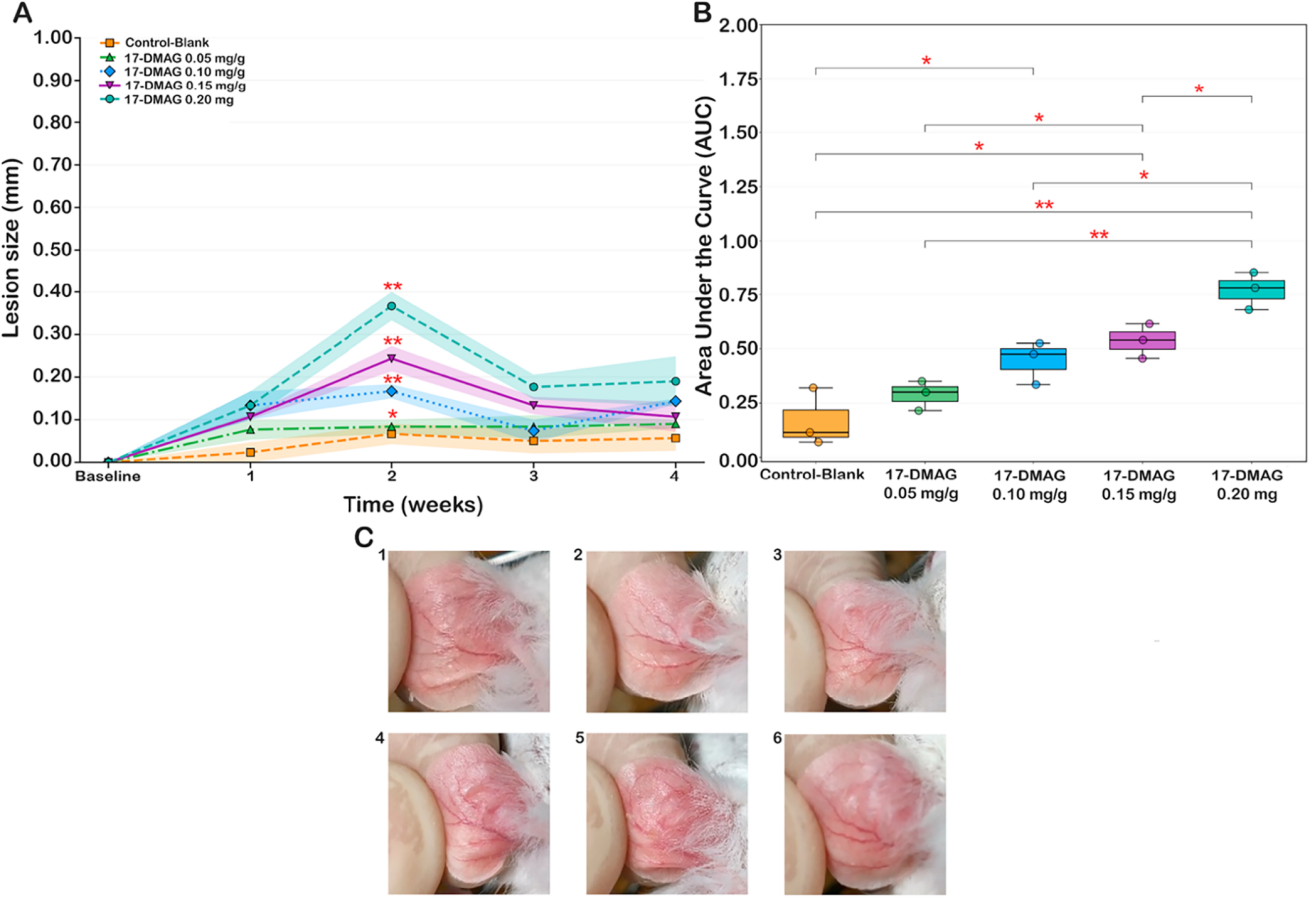
*In vivo* toxicity of topical 17-DMAG treatment formulated in CMC gel in BALB/c mice over 4 weeks. (A) Mean lesion size trajectory over the 4-week treatment period. The vehicle control group receiving CMC gel only (control-blank, *n* = 3) is shown as orange squares. Treatment groups include 17-DMAG at 0.05 mg/g (green triangles, *n* = 3), 0.10 mg/g (blue diamonds, *n* = 3), 0.15 mg/g (purple inverted triangles, *n* = 3), and 0.20 mg/g (green-blue circles, *n* = 3). Individual data points represent mean lesion size measured in millimeters at each time point, with shaded areas indicating the standard error of the mean (SEM) (One-Way ANOVA test, **p < 0.01, *p < 0.05); (B) box-and-whisker plots show the area under the curve (AUC) for lesion size over the 4-week treatment period in BALB/c mice treated with CMC gel alone or with 17-DMAG at 0.05, 0.10, 0.15, or 0.20 mg/g. Each point represents an individual animal (*n* = 3 per group). Statistical comparisons between groups were performed using Kruskal–Wallis; significant differences are indicated by red asterisks (*p < 0.05, **p < 0.01); (C) representative images of BALB/c mouse ears after 4 weeks of daily topical treatment with CMC gel formulations. (C1) Control blank (17-DMAG/7-DMAG-containing CMC hydrogel). (C2–C6) Ears treated with 17-DMAG incorporated into CMC gel at doses of 0.05 mg/g (C2), 0.10 mg/g (C3), 0.15 mg/g (C4), and 0.20 mg/g (C5, C6).

Cumulative local toxicity (AUC) increased with dose and correlated strongly with concentration (Spearman ρ = 0.9274, *p* < 0.001; Figure 4S). Overall group differences were significant (Kruskal–Wallis *p* = 0.016), with median AUC rising from 0.300 at 0.05 mg/g to 0.780 at 0.20 mg/g *versus* 0.115 in vehicle controls. Macroscopically, irritation remained mild (mainly slight hyperemia; Figure 4C) and tended to resolve over time (Figure 4A).

Histology showed mild inflammatory changes in manipulated ears, including in vehicle-treated animals, indicating that repeated topical manipulation contributed to local inflammation. Higher 17-DMAG concentrations were associated with more evident edema and inflammatory infiltrate (Figure 5). Ears of animals treated with drug-free gel (Figure 5B1) or gel containing 0.05 mg/g of 17-DMAG (Figure 5C1) exhibited mild inflammation, characterized by a few mononuclear cells, rare neutrophils, and epidermal thickening with increased keratinocytes. More pronounced inflammation was observed in animals treated with higher concentrations. In the 0.10 and 0.15 mg/g groups, histology showed mild inflammation with mononuclear cells, polymorphonuclear leukocytes, fibroblasts, edema, and epidermal thickening accompanied by crust formation and ulceration. Lesions from animals treated with 0.20 mg/g of 17-DMAG exhibited more pronounced edema (Figure 5F1). No histological abnormalities suggestive of systemic toxicity were observed in liver, spleen, or kidneys across treated groups (Figure 5).

**Figure 5:**
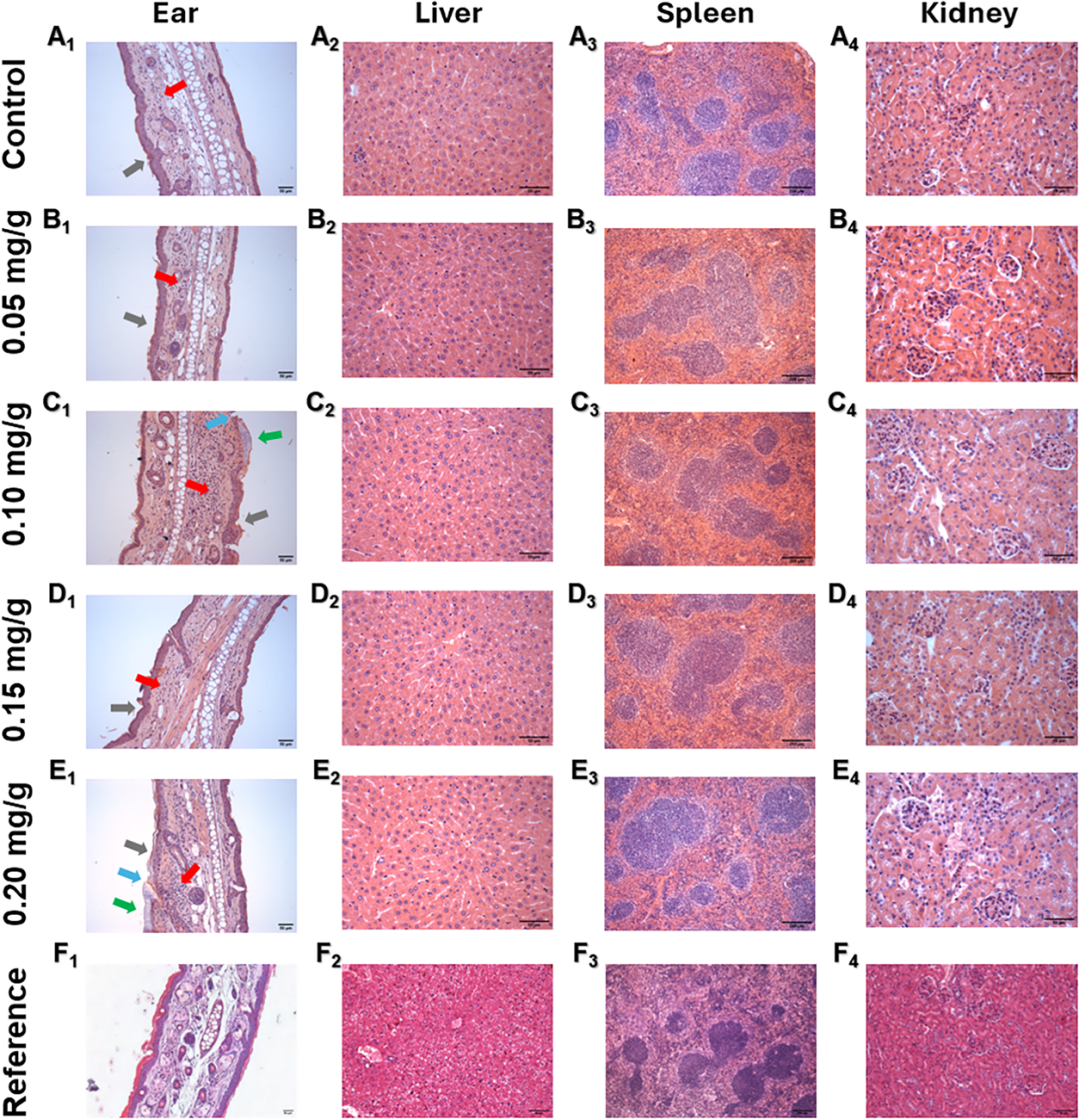
Histological analysis of tissues from BALB/c mice exposed to topical 17-DMAG gel. BALB/c mice were treated daily for 4 weeks with gel containing 0.05 mg/g (B), 0.10 mg/g (C), 0.15 mg/g (D), or 0.20 mg/g (E) of 17-DMAG, or with drug-free gel as a control (A). Reference animals were controls maintained with no manipulation (F). After euthanasia, ear (1), liver (2), spleen (3), and kidney (4) samples were collected and processed for H&E staining. Magnification: ear (20×), liver (40×), spleen (10×), and kidney (40×). Red arrows: inflammatory infiltrate with edema; green arrows: crust; blue arrows: ulcer; gray arrows: epidermal thickening with increased keratinocytes. Reference ear (F1): contralateral ear from mice exposed to drug-free gel. Reference liver (F2), kidney (F4), and spleen (F3) samples were obtained from healthy BALB/c mice.

Because animals in the control group treated with drug-free gel also exhibited inflammatory cell recruitment at the application site, we conducted an additional assay to determine whether the blank CMC gel contributed to local toxicity. Uninfected BALB/c mice were exposed daily for 4 weeks to gels containing 1% m/v CMC, 2% m/v CMC, Tegaderm alone, or were left unmanipulated. Histological analysis showed an inflammatory cell infiltration at the site in all manipulated groups (1% CMC, 2% CMC, and Tegaderm). These results indicate that daily topical manipulation itself contributed significantly to local inflammation, in addition to the mild dose-dependent toxicity associated with 17-DMAG (data not shown). Importantly, this inflammatory response was transient, gradually resolving after 3 weeks of treatment as observed in lesion size trajectory (Figure 4A).

### *In Vivo* Efficacy of Topical 17-DMAG

Based on tolerability, 0.05, 0.10, and 0.15 mg/g were tested for efficacy. Across the treatment period, mean lesion burden decreased in a dose-dependent manner: compared with blank CMC controls (0.40 ± 0.05 mm), 0.05 mg/g produced minimal change (−2.8%), whereas 0.10 and 0.15 mg/g reduced lesions by 27.0% and 29.5%, respectively (Figure 6A). Longitudinally, treatment effects emerged after week 2 and became clearer at weeks 3–4 (Figure 6B), showing statistical differences when comparing untreated control animals with those treated with either 0.10 or 0.15 mg/g of 17-DMAG-embedded CMC gels (Mann–Whitney U test). At week 4, both doses achieved similar median lesion sizes (0.30 mm), indicating an efficacy plateau above 0.10 mg/g dose. Effect size estimates supported large treatment effects at later time points (Figure 5S).

**Figure 6:**
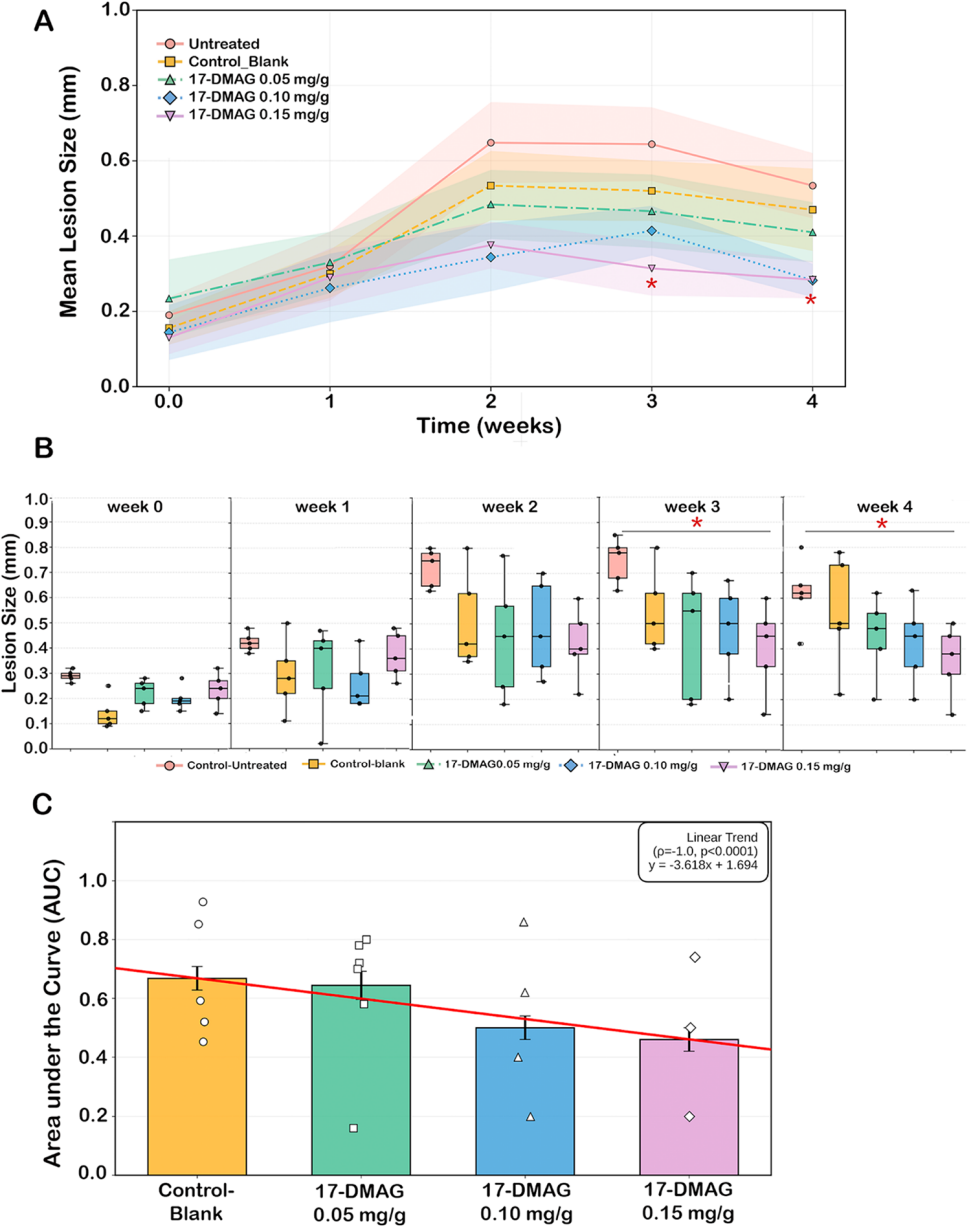
Efficacy of topical 17-DMAG gel in BALB/c mice infected with *L. braziliensis*. The untreated control group (untreated, *n* = 5) is shown as rose circles, and the vehicle control group receiving CMC gel only (control-blank, *n* = 5) is represented by yellow squares. Treatment groups include 17-DMAG CMC gel at 0.05 mg/g (green triangles, *n* = 5), 0.10 mg/g (blue diamonds, *n* = 5), and 0.15 mg/g (purple inverted triangles, *n* = 5). (A) Mean lesion size over the 4-week treatment period. Each data point represents the mean lesion diameter in millimeters, and shaded areas indicate the standard error of the mean (SEM); (B) Boxplot analysis showing the distribution of lesion sizes at each weekly time point (Weeks 0, 1, 2, 3, and 4) for BALB/c mice infected with *L. braziliensis* treated with different concentrations of 17-DMAG formulated in CMC gel. Statistical comparisons between groups were performed using Mann–Whitney U test; significant differences are indicated by red asterisks (*p < 0.05) in week 3 and 4. (C) Linear trend between the AUC values of the control-blank group (vehicle control, orange) and the groups treated with 17-DMAG CMC gel at 0.05 mg/g (green), 0.10 mg/g (blue), and 0.15 mg/g (purple). Bar heights represent the mean AUC for each group, with error bars indicating the standard error of the mean (SEM). The red line depicts the linear trend between dose and therapeutic response.

Independent-samples *t*-tests confirmed dose-dependent therapeutic efficacy of topical 17-DMAG with consistent results across control conditions. Relative to vehicle controls, both 0.10 and 0.15 mg/g formulations achieved statistical significance by week 3 (*p* = 0.026 and 0.036; Cohen’s *d* = 1.338 and 1.596), with large effect sizes maintained through week 4 (*d* = 1.006 and 0.979) (Figure 5S). Comparable results were observed *versus* untreated controls, reaching significance at week 4 (*p* = 0.0319 and 0.0362) with very large effect sizes (*d* = −1.640 and −1.589), supporting the CMC hydrogel as an inert vehicle. The minimal difference in week-4 effect sizes between doses (3.2%) indicates an efficacy plateau at 0.10 mg/g.

Consistent with these findings, AUC analysis showed a progressive reduction in cumulative lesion burden with increasing dose (Figure 6C). Linear regression demonstrated a strong dose–response relationship (Spearman ρ = −1.0, *p* < 0.0001; *R* ^2^ = 0.874), with each 0.05 mg/g dose increment associated with an estimated 0.09 mm decrease in mean lesion size.

Collectively, these analyses indicate that 0.15 mg/g produces the earliest measurable therapeutic effect, while both 0.10 and 0.15 mg/g achieve sustained efficacy from week 2 through the end of treatment.

Representative images were concordant with quantitative measures: untreated and vehicle-treated mice developed ulcerated lesions by week 4, whereas 0.10 and 0.15 mg/g groups showed smaller lesions and macroscopic signs of healing (Figure 7). Lymph node size remained similar across groups (Figure 7; data not shown). No changes in body weight, indicate that collar use did not interfere with feeding or hydration (data not shown)

**Figure 7:**
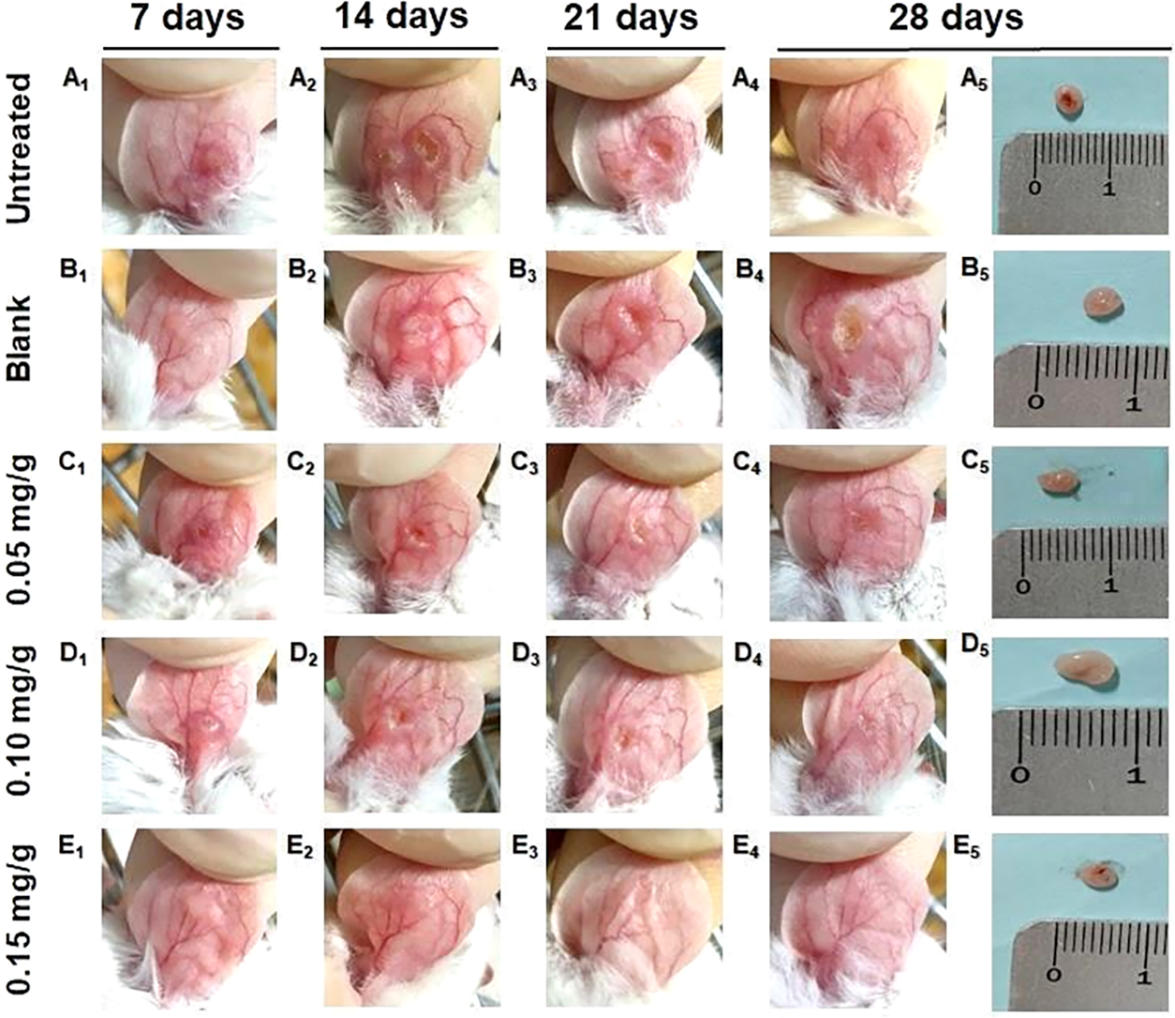
Representative images of ears and lymph nodes from BALB/c mice infected with *L. braziliensis* and treated with topical 17-DMAG CMC gel. BALB/c mice were infected in the ear with 5 × 10^5^ metacyclic *L. braziliensis* promastigotes. Control groups included untreated animals (A) and animals treated with CMC gel (blank) (B). Treatment groups received daily topical application for 4 weeks (images shown at days 7, 14, 21, and 28) of CMC gel containing 0.05 mg/g (C), 0.10 mg/g (D), or 0.15 mg/g (E) 17-DMAG. After completion of treatment, ears and retroauricular lymph nodes were collected for analysis.

### Kaplan–Meier Analysis of Healing

Kaplan–Meier analysis was used to evaluate lesion healing kinetics over the 4-week treatment period in *L. braziliensis*–infected mice (Figure 8). Lesion progression for all animals throughout the follow-up period is shown in Figure 6S. Healing was absent in both vehicle-treated and untreated control groups throughout the study, confirming the chronic nature of lesions in this model (Figure 8A). In contrast, topical 17-DMAG in CMC gel induced clear, dose-dependent healing responses, with onset observed as early as week 1 in treated groups (Figure 8A). Among the tested formulations, 17-DMAG at 0.10 mg/g demonstrated superior efficacy and fastest healing kinetics: 60% of animals achieved complete lesion resolution by week 2, increasing to 80% by week 3. This level of healing was sustained through week 4, indicating a robust and durable therapeutic response.

**Figure 8:**
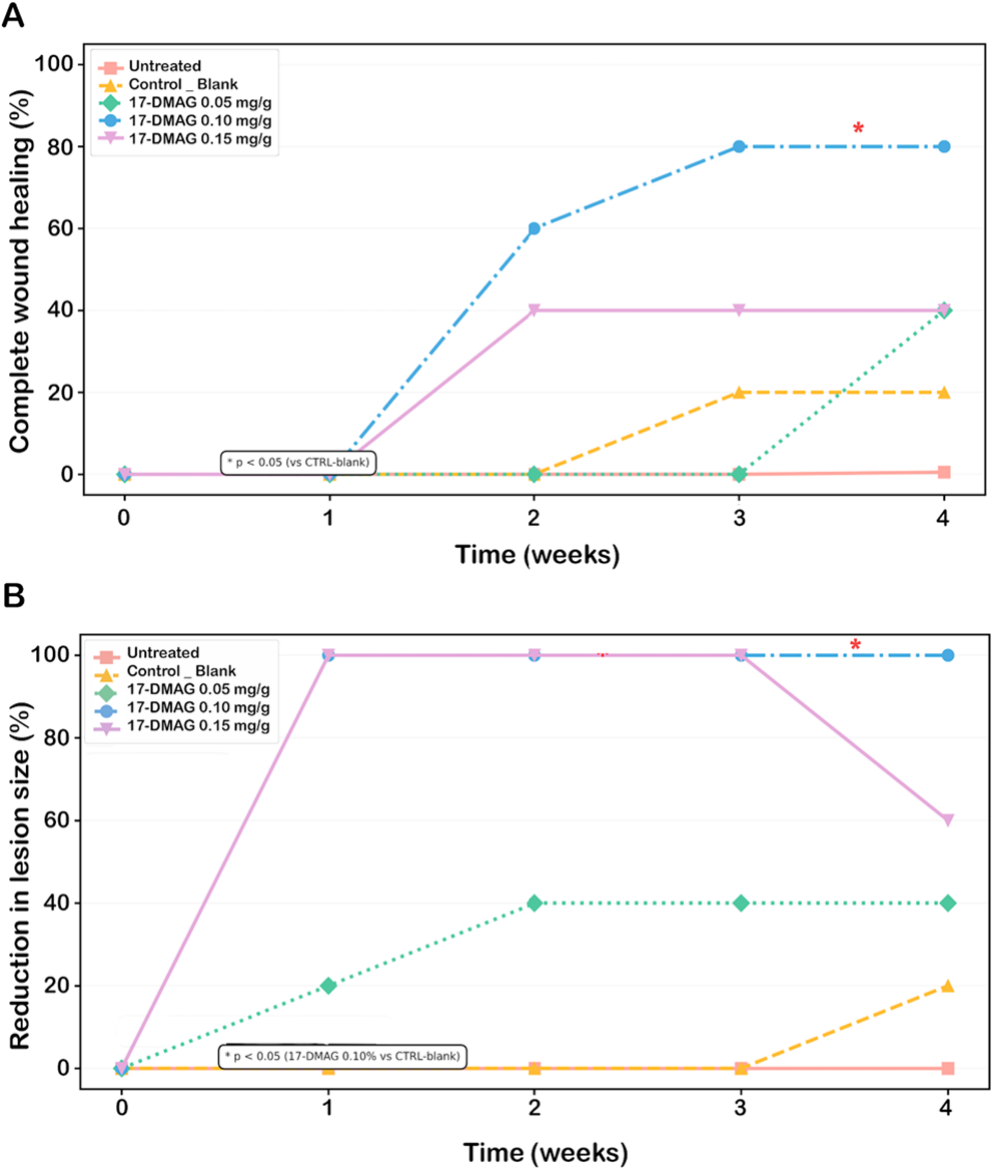
Kaplan–Meier healing curves showing the cumulative percentage of lesion outcomes in *L. braziliensis*–infected mice treated with topical 17-DMAG formulations. Kaplan–Meier analysis depicting the cumulative percentage of *L. braziliensis*-infected mice achieving complete lesion healing (A) or reduction in lesion size (B) across weekly intervals (weeks 0–4). Mice were topically treated over the 4 weeks period with 17-DMAG CMC gel at 0.05 mg/g (green diamonds, *n* = 5), 0.10 mg/g (blue circles, *n* = 5), and 0.15 mg/g (purple inverted triangles, *n* = 5) as described in Figure 6. The untreated control group (untreated, *n* = 5) is shown as rose squares, and the vehicle control group receiving CMC gel (control blank, *n* = 5) is represented by orange triangles. Differences among Kaplan–Meier’s curves were considered significant when *p* < 0.05 (Fisher’s exact test, *p = 0.0476).

## Discussion

CL remains a neglected tropical disease for which most available treatments rely on parenteral administration, resulting in patient discomfort, poor adherence, high costs, and suboptimal outcomes.^32^ Given these limitations, alternative therapeutic strategies, particularly topical formulations, have been actively explored.^32^ Topical delivery offers a patient-friendly route of administration and enables high drug concentrations at the lesion site, potentially improving efficacy and adherence.^33,34^ Despite this promise, no topical strategy has yet been successfully translated into routine clinical practice.

In this study, we evaluated the toxicity and therapeutic efficacy of a topical CMC gel formulation containing 17-DMAG, a compound with well-established antileishmanial activity. Previous *in vitro* studies demonstrated that 17-DMAG is highly active against *L. braziliensis* with low toxicity toward mammalian cells, yielding a high selectivity index (432) against intracellular parasites.^18^ Systemic administration of 17-DMAG has also been shown to promote wound healing and eliminate parasites from both lesion sites and draining lymph nodes.^18^

The low *in vitro* cytotoxicity observed in HaCaT keratinocytes (7.6 ± 0.2 μM) supports the translational potential of 17-DMAG as a topical therapy for CL. HaCaT cells are widely used to predict skin tolerance, and the favorable safety profile observed here provided a rationale for advancing 17-DMAG to *in vivo* evaluation.^35,36^ Consistently, limited toxicity was also observed in THP-1 human macrophages (7.1 ± 0.2 μM), a relevant model of the primary host cell for *Leishmania*, indicating that therapeutic activity can be achieved without compromising host-cell viability. Together, these findings supported the development and testing of 17-DMAG as a locally administered therapy aimed at minimizing systemic exposure while preserving efficacy. Similar predictive relationships between *in vitro* selectivity and topical safety have been reported for other compounds, including dapsone^22^ and cinnamaldehyde.^37^ In this context, topical 17-DMAG could work as a complementary approach to established systemic treatments such as pentavalent antimonials, amphotericin B, and miltefosine.^11,12^ This strategy is in line with recent clinical recommendations from the Drugs for Neglected Diseases initiative (DNDi) and the Brazilian Ministry of Health, which advocate for therapeutic regimens that combine local and systemic interventions for the management of uncomplicated lesions while reducing overall systemic drug exposure.^25^

CMC gels were selected as delivery vehicles due to their biocompatibility, biodegradability, low immunogenicity, and established use in topical drug delivery.^38^ Stability studies demonstrated that the CMC gel preserved the chemical integrity of 17-DMAG at 4 and 25 °C for 90 days, whereas significant degradation occurred at 37 °C. These findings are consistent with known temperature-dependent instability of 17-DMAG in aqueous solutions and highlight temperature as a critical determinant of formulation stability.^39–41^ Similar temperature-driven degradation has been reported for other topical gels, such as tetracycline formulations.^42^ Given that CL predominantly affects populations in resource-limited settings, formulation strategies that ensure stability at ambient or elevated temperatures will be essential. Our results therefore emphasize the need for further optimization to enhance molecular stability during future development studies for future human testing. The release profile of 17-DMAG from the CMC gel followed a diffusion-controlled mechanism, with approximately 45% of the drug released within 24 h. Higuchi modeling predicted release onset after an initial lag phase (∼0.36 h), consistent with the hydration dynamics of polymeric matrices.^20,43–46^ Thus, the CMC gel’s release profile demonstrated diffusion-controlled behavior, supporting local drug permeation into the cutaneous layers. This permeation is essential for the drug to encounter and eliminate intracellular amastigotes in the dermis, as reflected in the significant reduction of lesion size in treated mice. Comparable release profiles have been described for other antimicrobial agents formulated in polymeric gels, including levofloxacin from chitosan gels (∼70% released in 60 h)^47^ and acyclovir from β-cyclodextrin-conjugated CMC matrices (60% in 18 h and complete release at 24 h).^48^

Dual-action therapies represent an attractive strategy for CL, combining direct antiparasitic effects with modulation of host inflammatory responses. Previous studies showed that systemic 17-DMAG reduced inflammatory cytokines, including IL-6, IFN-γ, TNF, and IL-10, suggesting immunomodulatory activity in addition to parasite killing.^18^ In the present study, the CMC hydrogel incorporating 17-DMAG was applied daily for 4 weeks, in line with our diffusion-controlled release profile and pharmacokinetics studies in humans and dogs.^49–51^ In the present study, *in vivo* safety assays revealed mild and transient local inflammation across all manipulated groups, including vehicle controls, indicating that daily topical manipulation contributed to inflammatory responses. Although ear thickness increased in a dose-dependent manner, these changes were reversible and resolved by the third week of treatment. Such transient discrete irritation is common in topical toxicity assays and often overestimates human responses.^52,53^ These findings support the local safety of 17-DMAG CMC formulations.

Topical delivery of 17-DMAG (0.15 mg/g) resulted in approximately 50% lesion size reduction compared with untreated controls, an effect comparable to other experimental topical formulations, including BC-DETC, 8-hydroxyquinoline, and paromomycin-based gels.^54–57^ However, many published studies lack standardized parasitological or toxicity end points, limiting direct cross-study comparisons. In this study, reinforcing formulation effect, time-to-healing analysis revealed clear differences in therapeutic kinetics between treated and control groups. The absence of lesion resolution in vehicle-treated and untreated animals throughout the 4-week period confirms the chronic nature of *L. braziliensis* infection in this model. In contrast, topical 17-DMAG induced a dose-dependent and progressive healing response, with clinical lesion resolution detected early during treatment. Among the tested formulations, 17-DMAG at 0.10 mg/g showed the most favorable profile, combining rapid onset of healing with sustained efficacy. Most animals achieved complete lesion resolution by week 2, with maximal healing reached by week 3 and maintained through week 4, indicating a durable therapeutic effect. These findings highlight the importance of dose optimization for topical delivery and support 17-DMAG gel as a promising noninvasive approach for cutaneous leishmaniasis.

In this context, our results further support Hsp90 inhibition as a viable topical strategy for CL, demonstrating efficacy without detectable systemic toxicity. Next steps will focus on optimizing the formulation for better stability of 25 °C, a critical requirement for transport and storage in remote tropical regions. Future studies will also explore therapy with 17-DMAG gel alongside systemic standard-of-care drugs.

## Conclusion

Taken together, these results demonstrate that topical administration of 17-DMAG gel at 0.10 and 0.15 mg/g effectively controls experimental *L. braziliensis* infection in BALB/c mice, with the strongest therapeutic effects observed after 4 weeks of treatment. These findings support 17-DMAG gel as a promising noninvasive approach for cutaneous leishmaniasis. However, the lack of reduction in lymph node size indicates that topical application should be considered as a complementary strategy alongside systemic therapies, such as pentavalent antimonials or miltefosine, to focus on accelerated wound healing while systemic agents target parasites in deeper tissues. This strategy aims to address the challenges of severe systemic toxicity and the invasive nature of current regimens by providing localized healing and reducing the systemic drug burden required for a complete cure.

## Methods

### Cell-Line Culture

Human immortalized keratinocytes (HaCaT) were cultured in complete DMEM [DMEM (Capricorn, Ebsdorfergrund, Hesse, Germany)] supplemented with 10% heat-inactivated fetal bovine serum [FBS (Gibco, Waltham, MA, USA)], 2 g/L sodium bicarbonate, 22.8 mM HEPES (Sigma-Aldrich, St. Louis, MO, USA), and 0.2% ciprofloxacin (Fresenius Kabi Brasil Ltd.a, Aquiraz, CE, Brazil). The human monocyte cell line THP-1 was maintained in complete RPMI [RPMI (Gibco, Waltham, MA, USA)] supplemented with 10% FBS, 22.8 mM HEPES, 27 mM sodium bicarbonate, 2 mM l-glutamine, and 2 mM sodium pyruvate (all from Sigma-Aldrich), and 10 μg/mL ciprofloxacin (Fresenius Kabi Brasil Ltd.a, São Paulo, Brazil). Both cell lines were cultured at 37 °C in a humidified atmosphere with 5% CO_2_; HaCaT cells were subcultured at 80–85% confluence using 0.05% trypsin–EDTA (Sigma-Aldrich) and counted in a Neubauer chamber, while THP-1 cells were used up to passage 10.

### *In Vitro* Cytotoxicity

Cytotoxicity of 17-DMAG (Fermentek Ltd., Jerusalem, Israel) was evaluated in HaCaT cells seeded at 3 × 10^4^ cells/well in 96-well plates containing 200 μL complete high-glucose DMEM. After 24 h, cells were exposed to increasing concentrations of 17-DMAG for 48 h, followed by addition of 10% (v/v) Alamar Blue reagent (Invitrogen, Carlsbad, CA, USA) and incubation for an additional 24 h. Absorbance was measured at 570 and 600 nm using a SPECTRAmax 340PC spectrophotometer (Molecular Devices, San José, CA, USA). For THP-1 assays, 5 × 10^4^ cells/well were differentiated with 100 nM phorbol 12-myristate 13-acetate (PMA; Sigma-Aldrich) for 24 h, washed with 0.9% NaCl (Farmace, Barbalha, CE, Brazil), and incubated with 17-DMAG (0.78–100 μM) for 48 h before Alamar Blue addition. CC_50_ values were calculated by nonlinear regression (variable slope) using GraphPad Prism v10.4.0 (GraphPad Software, San Diego, CA, USA).

### Preparation and Characterization of 17-DMAG–Loaded CMC Gels

A 1% or 2% (w/w) CMC (Delaware, Porto Alegre, RS, Brazil) dispersion was prepared by slowly adding the polymer to deionized water under continuous magnetic stirring at 37 °C until a homogeneous gel was formed. 17-DMAG loaded formulations were prepared using the same procedure, replacing the aqueous phase with previously prepared 17-DMAG stock solutions to obtain final concentrations of 0.30, 0.25, 0.20, 0.15, 0.10, or 0.05 mg/g. All formulations were freshly prepared prior to *in vivo* use. The macroscopic properties, including color and viscosity, were evaluated over 90 days (1, 7, 14, 21, 28, 60, and 90 days) of storage at 4 °C, 25 °C, or 37 °C. A white hydrogel without 17-DMAG was used as the control.

### Stability of 17-DMAG-Loaded CMC Gels

Formulations containing 0.30, 0.25, 0.20, and 0.15 mg/g of 17-DMAG were stored in a conical centrifuge tube protected from direct light for 90 days at 4 °C in a refrigerator (Consul, Joinville, SC, Brazil), at a medium room temperature of 25 °C, and 37 °C in an incubator (Thermo Fisher, Waltham, MA, EUA).

On days 1, 7, 14, 21, 28, 60 and 90, the samples were analyzed for their macroscopic characteristics, such as color and apparent viscosity, by naked eye. In addition, an aliquot was diluted with distilled water to bring the final concentration within the analytical range. After centrifugation (125*g*, 2 min), 200 μL of the supernatant was transferred, in triplicate, to a 96-well plate together with calibration standards (0.05–0.0001 mg/mL) (Figure 1S) and an internal control (0.30 mg/mL). Absorbance was recorded at 340 nm using a SPECTRAmax-340PC reader, and concentrations were calculated from the daily calibration curve.

### *In Vitro* Drug Release

*In vitro* release of 17-DMAG was assessed using Franz diffusion cells (DHC-6T, Logan Instruments, Somerset, NJ, USA) fitted with dialysis membranes (MWCO 6000–8000 Da). Five milligrams of gel containing 0.33 mg/g 17-DMAG were applied to the donor compartment (*n* = 3), while the receptor chamber contained 10 mL phosphate-buffered saline (PBS, pH 7.4) (Invitrogen, Carlsbad, CA, USA) maintained at 37 °C under magnetic stirring (300 rpm). At predefined time points up to 24 h, the entire receptor phase was collected, frozen at −20 °C, and replaced with fresh PBS. Quantification was performed by HPLC (Shimadzu LC-20A, Kyoto, Japan) using a C18 reversed-phase column under isocratic elution with acetonitrile (27%), methanol (27%), ultrapure water (46%), and trifluoroacetic acid (0.05%) (Sigma-Aldrich), at a flow rate of 1 mL/min and detection at 340 nm. Calibration curves were generated from serial dilutions of 17-DMAG (0.625–10 μg/mL) using Empower software v3 (Auckland, North Island, New Zealand) (Figure 2S).

### Parasite Maintenance

*L. braziliensis* (MHOM/BR/01/BA788) promastigotes were isolated from infected BALB/c mice and cultured in Schneider’s Insect Medium (Sigma-Aldrich) supplemented with 20% FBS (Gibco) and 50 μg/mL gentamicin (Gibco) at 24 °C for up to seven passages. Stationary-phase parasites were washed, counted, and adjusted to experimental concentrations. For *in vivo* assays, parasites were enriched for metacyclic forms by incubation with 1 mg/mL *Bauhinia purpurea* lectin (Vector Laboratories, Newark, CA, USA) for 30 min at room temperature, followed by washing and adjustment to 5 × 10^6^ parasites/20 μL for ear inoculation, as described by Cruz et al.^18^

### Animals and Ethics

Female BALB/c mice (6–8 weeks old) were housed at the Animal Care Facility of the Gonçalo Moniz Institute (IGM-FIOCRUZ-BA) with free access to food and water. All procedures were approved by the Institutional Animal Care and Use Committee (protocols 007/2015 and 007/2020).

### *In Vivo* Toxicity

For toxicity evaluation, healthy mice received daily topical applications of 17-DMAG gels (0.05, 0.10, 0.15, or 0.20 mg/g) or blank 2% CMC gel for 4 weeks. Formulations were applied to the ear and protected with Elizabethan collars and Tegaderm adhesive dressings (Vital Derme, Maringá, PR, Brazil). Ear thickness and local reactions were assessed weekly using a Kroeplin analog caliper (±0.1 mm). Animals were euthanized with thiopental (50 mg/kg, intraperitoneally), and ears, liver, spleen, and kidneys were collected for histopathology.

### *In Vivo* Efficacy

For efficacy studies, mice were infected with 5 × 10^5^ stationary-phase *L. braziliensis* promastigotes and treated 3 weeks later with daily topical applications of 17-DMAG gels (0.05, 0.10, or 0.15 mg/g) for 4 weeks. Control-blank-gel-treated and untreated infected animals served as controls. Lesion progression was monitored weekly by measuring ear thickness relative to the contralateral ear. At the end of treatment, ears and retroauricular lymph nodes were collected.

### Histopathology

Tissues from healthy and *L. braziliensis*-infected BALB/c mice were fixed for 48 h in acid formalin solution (5% glacial acetic acid, 10% formaldehyde, 85% absolute ethanol; Sigma-Aldrich), paraffin-embedded, sectioned, and stained with hematoxylin and eosin for light microscopy evaluation.

### Statistical Analysis

Statistical analyses were performed using GraphPad Prism v9.0. Data distribution guided the use of parametric or nonparametric tests. Student’s *t*-test or one-way ANOVA with Tukey’s post hoc test were applied to Gaussian data, while Mann–Whitney or Kruskal–Wallis tests were used otherwise. Longitudinal toxicity and efficacy data were analyzed by area under the curve, Spearman correlation, Cohen’s *d* effect size, and Kaplan–Meier time-to-healing analysis. Healing was defined as complete lesion resolution with normal skin appearance, and clinical improvement as ≥ 25% lesion size reduction from baseline.^58^

## Supplementary Material



## Notes

Physicochemical characterization and analytical validation of 17-DMAG (UV–Vis and HPLC calibration curves); macroscopic and quantitative stability studies of 17-DMAG-loaded CMC hydrogels under different storage temperatures; heatmap analysis of drug retention; dose–response correlation analysis of topical toxicity (AUC); pairwise statistical analyses of lesion size progression; and representative macroscopic images of lesion evolution in treated and control animals (PDF)

## Abbreviations

AUC: area under the curve
BMDM or BMMΦ: bone marrow–derived macrophages
B.O.D.: biological oxygen demand incubator
CC_50_: 50% cytotoxic concentration
CI: confidence interval
CL: cutaneous leishmaniasis
CMC: carboxymethylcellulose
DMEM: Dulbecco’s modified Eagle medium
DNDi: Drugs for Neglected Diseases initiative
EDTA: ethylenediaminetetraacetic acid
EGFR: epidermal growth factor receptor
FBS: fetal bovine serum
H&E: hematoxylin and eosin
HaCaT: human immortalized keratinocyte cell line
HEPES: 4-(2-hydroxyethyl)-1-piperazineethanesulfonic acid
HPLC: high-performance liquid chromatography
Hsp90: heat shock protein 90
IFN-γ: interferon gamma
IL: interleukin
IP: intraperitoneal
kDa: kilodalton
*Lb*: *Leishmania braziliensis*
μg: microgram
μL: microliter
μM: micromolar
MWCO: molecular weight cutoff
PMA: phorbol 12-myristate 13-acetate
PBS: phosphate-buffered saline
PAHO: Pan American Health Organization
RPMI: Roswell Park Memorial Institute medium
SD: standard deviation
SEM: standard error of the mean
SI: selectivity index
TFA: trifluoroacetic acid
THP-1: human monocytic leukemia cell line
TNF: tumor necrosis factor
VL: visceral leishmaniasis
WHO: World Health Organization
