# Supplementary material for: Development of a 17-DMAG-Loaded Carboxymethylcellulose Gel for In Vivo Treatment of Cutaneous Leishmaniasis
Source: ACS Omega. 2026 May 5;11(19):28432–43. doi: 10.1021/acsomega.6c00447 (PMC13191516; doi:10.1021/acsomega.6c00447)
Supplement: Supplementary file 1 [file ao6c00447_si_001.pdf]

Development of a 17-DMAG-loaded carboxymethylcellulose gel for *in vivo* treatment of Cutaneous Leishmaniasis

Kercia Pinheiro Cruz[1]; Mariana Rolemberg Gueudeville Silveira[1]; Igor Rolemberg Gueudeville Silveira[1]; Jade Liz Ferreira Mendes Souza[1]; Marina Faillace de Amorim[1]; Alan Gualberto de Souza de Freitas de Pinho[1;] Ana Luiza de Jesus Cordeiro[1]; Izabella Gouveia Oliveira[1]; Isadora dos Santos Lima[1]; Claudia Ida Brodskyn[1]; Juliana Perrone Bezerra de Menezes[1,2]; Deborah Bittencourt Motte[1,2,3]; Henrique Rodrigues Marcelino[4]; Fabio Rocha Formiga[5,6]; Washington Luis Conrado dos Santos[7,8]; Thamires Quadros Froes[1]; Patricia Sampaio Tavares Veras[1,2*]

[1]Laboratory of Host-Parasite Interaction and Epidemiology, Gonçalo Moniz Institute, Fiocruz-Bahia, Salvador 40296-710, Bahia, Brazil;

[2]National Institute of Science and Technology of Tropical Diseases (INCT-DT), National Council for Scientific Research and Development (CNPq), Salvador, Brazil;

[3]Department of Preventive Veterinary Medicine and Animal Production, School of Veterinary Medicine and Animal Science, Federal University of Bahia, Salvador 40170-110, Bahia, Brazil;

[4]Department of Medicines, College of Pharmacy, Federal University of Bahia, Salvador/BA, Brazil, 40170-115;

[5]Aggeu Magalhães Institute, Oswaldo Cruz Foundation (FIOCRUZ), 50670-420, Recife, PE, Brazil;

[6]Faculty of Medical Sciences, University of Pernambuco, 50100-130, Recife, PE, Brazil;

[7]Laboratory of Structural and Molecular Pathology, Gonçalo Moniz Institute, Oswaldo Cruz Foundation (FIOCRUZ), Salvador, Brazil;

[8]Department of Pathology and Forensic Medicine, Bahia Medical School, Federal University of Bahia, Salvador 40110-906, Bahia, Brazil

*Corresponding author: patricia.veras@fiocruz.br (PSTV)

Table of Contents

Figure 1S: Calibration curve of 17-DMAG determined by UV-Vis. The calibration curve was obtained using 17-DMAG standard solutions ranging from 0.05 to 0.0001 mg/mL. 4

Figure 2S: Calibration curve of 17-DMAG determined by HPLC. The calibration curve was obtained using 17-DMAG standard solutions ranging from 0.625 to 10 µg/mL. 5

Figure 3S. Stability of 17-DMAG–loaded CMC hydrogels under different storage conditions.(A) Macroscopic aspect of 17-DMAG incorporated into CMC hydrogel after 90 days of storage at 4 °C, 25 °C, or 37 °C. 17-DMAG was incorporated into the hydrogel at concentrations of 0.15 mg/g (yellow square), 0.20 mg/g (triangle green), 0.25 mg/g (inverted triangle red), and 0.30 mg/g (purple diamond) (w/w) and stored at 4 °C, 25 °C, or 37 °C (C). As a control, a white hydrogel without 17-DMAG (transparent circle) was prepared using 2% CMC in distilled water. Stability was evaluated over 90 days, and at the end of the experiment, the macroscopic appearance of each formulation was documented by imaging. (B) Drug content of 17-DMAG in CMC hydrogel formulations after 90 days of storage at different temperatures. The heatmap shows the percentage of 17-DMAG retained on day 90 relative to day 1 for all tested formulations and storage conditions. The color scale reflects the stability profile: green indicates high retention (≥90%), yellow-green indicates moderate retention (70–90%), and orange-to-red indicates low retention (<70%). Each cell displays the mean retention rate, calculated as (day 90 concentration / day 1 concentration) × 100, providing a direct measure of formulation stability under each storage condition. Rows correspond to CMC hydrogels containing 17-DMAG at 0.15, 0.20, 0.25, and 0.30 mg/g (w/w), and columns correspond to storage temperatures of 4 °C, 25 °C, and 37 °C. 7

Figure 4S: Dose–response correlation analysis of CMC hydrogels containing 17-DMAG at different concentrations with lesion size. Spearman rank correlation analysis was performed to assess the relationship between 17-DMAG concentration in CMC and cumulative dermal toxicity (expressed as area under the curve, AUC) in uninfected BALB/c mice over a 4-week treatment period. Each data point represents an individual animal treated with either the vehicle control (CTRL-blank) or CMC hydrogel containing 0.05, 0.10, 0.15, or 0.20 mg/g of 17-DMAG. The x-axis shows the 17-DMAG concentration, while the y-axis displays the cumulative lesion burden calculated as AUC. Data points are color-coded by group: orange circles (CTRL-blank), green squares (0.05 mg/g), blue diamonds (0.10 mg/g), purple triangles (0.15 mg/g), and inverted purple

triangles (0.20 mg/g). The red solid line represents the linear regression fit, with the equation $y = 2.910x + 0.151$. Statistical significance is indicated by Spearman's rank correlation coefficient ($\rho = 0.927$, $p < 0.001$). 8

Figure 5S: Pairwise independent t-test analyses comparing 17-DMAG treatments to the untreated control at each time point. This figure presents the p-value distributions across the 4-week treatment period for CMC hydrogels containing 0.05, 0.10, 0.15, or 0.20 mg/g of 17-DMAG compared with untreated controls. The red and blue dashed lines indicate Bonferroni-corrected significance thresholds at $\alpha = 0.05$ and $\alpha = 0.01$, respectively. (A) Independent t-test comparisons of lesion sizes in mice treated with CMC hydrogel containing 0.05 mg/g 17-DMAG versus untreated controls across all time points. No statistically significant differences were observed at any time point (week 0: $p = 0.7106$; week 1: $p = 0.9370$; week 2: $p = 0.2797$; week 3: $p = 0.2337$; week 4: $p = 0.3228$). (B) Independent t-test comparisons of lesion sizes in mice treated with CMC hydrogel containing 0.10 mg/g 17-DMAG vs. untreated controls. No statistically significant differences were detected through week 2 (week 0: $p = 0.6138$; week 1: $p = 0.6655$; At weeks 2 and 3, the p-value approached significance ($p = 0.0627$ and $p = 0.0876$, respectively), and by Week 4, the difference reached statistical significance at the $\alpha = 0.05$ level ($p = 0.0319$). (C) Independent t-test comparisons of lesion sizes in mice treated with CMC hydrogel containing 0.15 mg/g 17-DMAG vs. untreated controls. Between weeks 0 and 2, no significant differences were observed (week 0: $p = 0.3838$; week 1: $p = 0.8095$; week 2: $p = 0.0600$). At week 3, the strongest effect was detected ($p = 0.0265$), and by week 4, the treatment again reached statistical significance ($p = 0.0362$) at the $\alpha = 0.05$ level. 9

Figure 6S: Macroscopic appearance of ear lesion progression in BALB/c mice infected with *L. braziliensis*. Pannels correspond to images obtained from each animal topically treated with CMC hydrogels containing 0.05, 0.10, 0.15 mg/g of 17-DMAG for four weeks (28 days), as well as control groups, untreated and blank treated with 17-DMAG-free CMC hydrogel. Images were acquired each seven days until 28 days of follow-up**.** 14

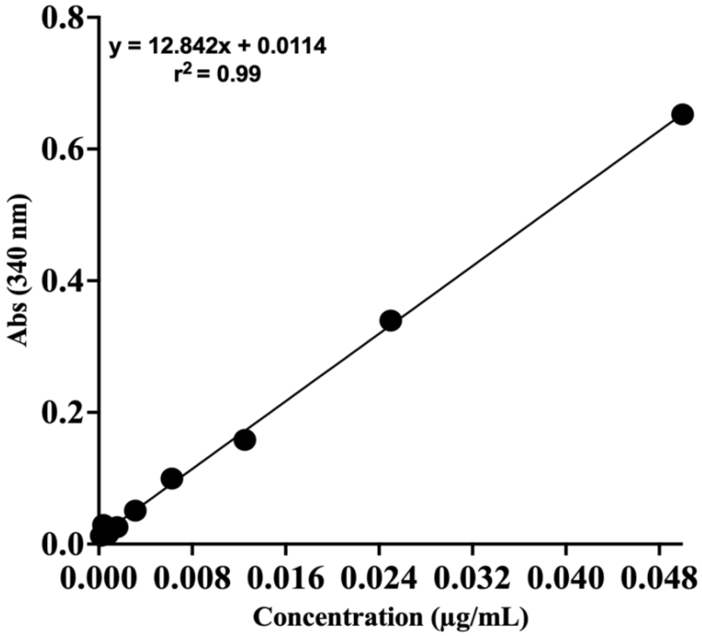

Figure 1S: Calibration curve of 17-DMAG determined by UV-Vis. The calibration curve was obtained using 17-DMAG standard solutions ranging from 0.05 to 0.0001 mg/mL.

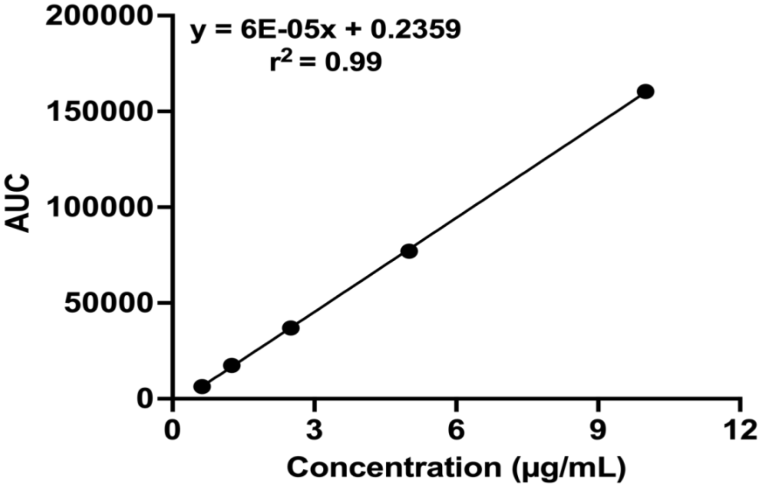

Figure 2S: Calibration curve of 17-DMAG determined by HPLC. The calibration curve was obtained using 17-DMAG standard solutions ranging from 0.625 to 10 µg/mL.

A

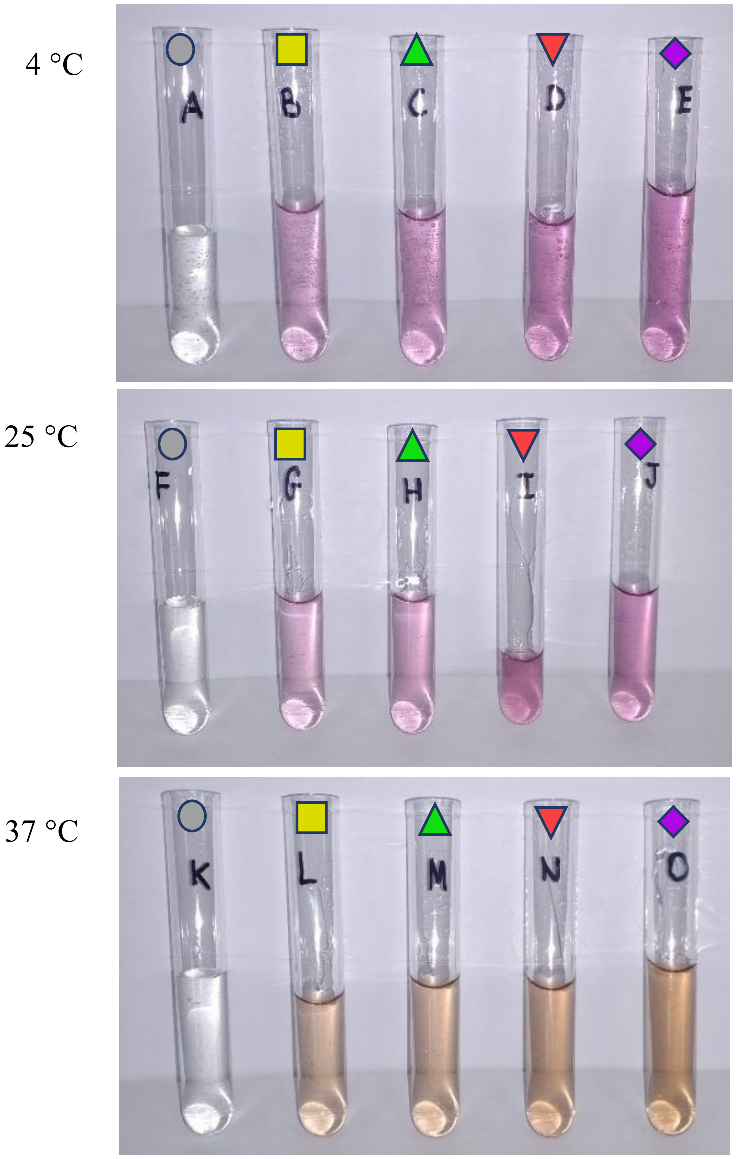
4 °C
A
B
C
D
E
25 °C
F
G
H
I
J
37 °C
K
L
M
N
O

B

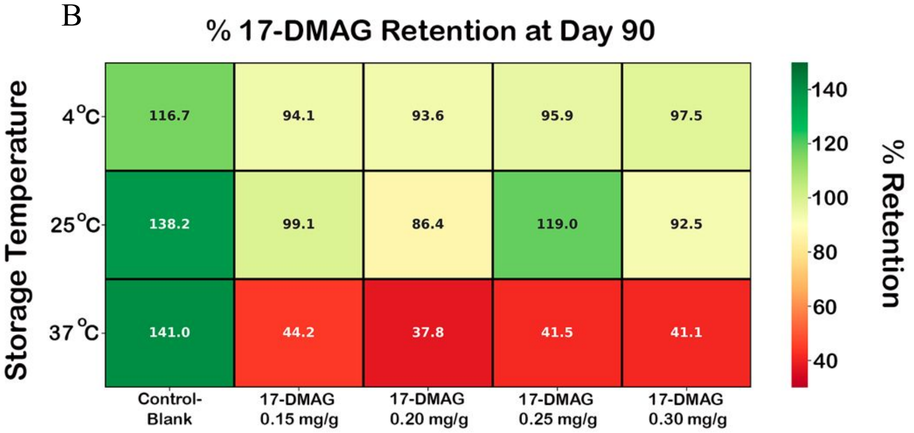
% 17-DMAG Retention at Day 90
Storage Temperature
4°C
25°C
37°C
116.7
94.1
93.6
95.9
97.5
138.2
99.1
86.4
119.0
92.5
141.0
44.2
37.8
41.5
41.1
Control-Blank
17-DMAG 0.15 mg/g
17-DMAG 0.20 mg/g
17-DMAG 0.25 mg/g
17-DMAG 0.30 mg/g
% Retention
140
120
100
80
60
40

Figure 3S. Stability of 17-DMAG–loaded CMC hydrogels under different storage conditions.(A) Macroscopic aspect of 17-DMAG incorporated into CMC hydrogel after 90 days of storage at 4 °C, 25 °C, or 37 °C. 17-DMAG was incorporated into the hydrogel at concentrations of 0.15 mg/g (yellow square), 0.20 mg/g (triangle green), 0.25 mg/g (inverted triangle red), and 0.30 mg/g (purple diamond) (w/w) and stored at 4 °C, 25 °C, or 37 °C (C). As a control, a white hydrogel without 17-DMAG (transparent circle) was prepared using 2% CMC in distilled water. Stability was evaluated over 90 days, and at the end of the experiment, the macroscopic appearance of each formulation was documented by imaging. **(B)** Drug content of 17-DMAG in CMC hydrogel formulations after 90 days of storage at different temperatures. The heatmap shows the percentage of 17-DMAG retained on day 90 relative to day 1 for all tested formulations and storage conditions. The color scale reflects the stability profile: green indicates high retention (≥90%), yellow-green indicates moderate retention (70–90%), and orange-to-red indicates low retention (<70%). Each cell displays the mean retention rate, calculated as (day 90 concentration / day 1 concentration) × 100, providing a direct measure of formulation stability under each storage condition. Rows correspond to CMC hydrogels containing 17-DMAG at 0.15, 0.20, 0.25, and 0.30 mg/g (w/w), and columns correspond to storage temperatures of 4 °C, 25 °C, and 37 °C.

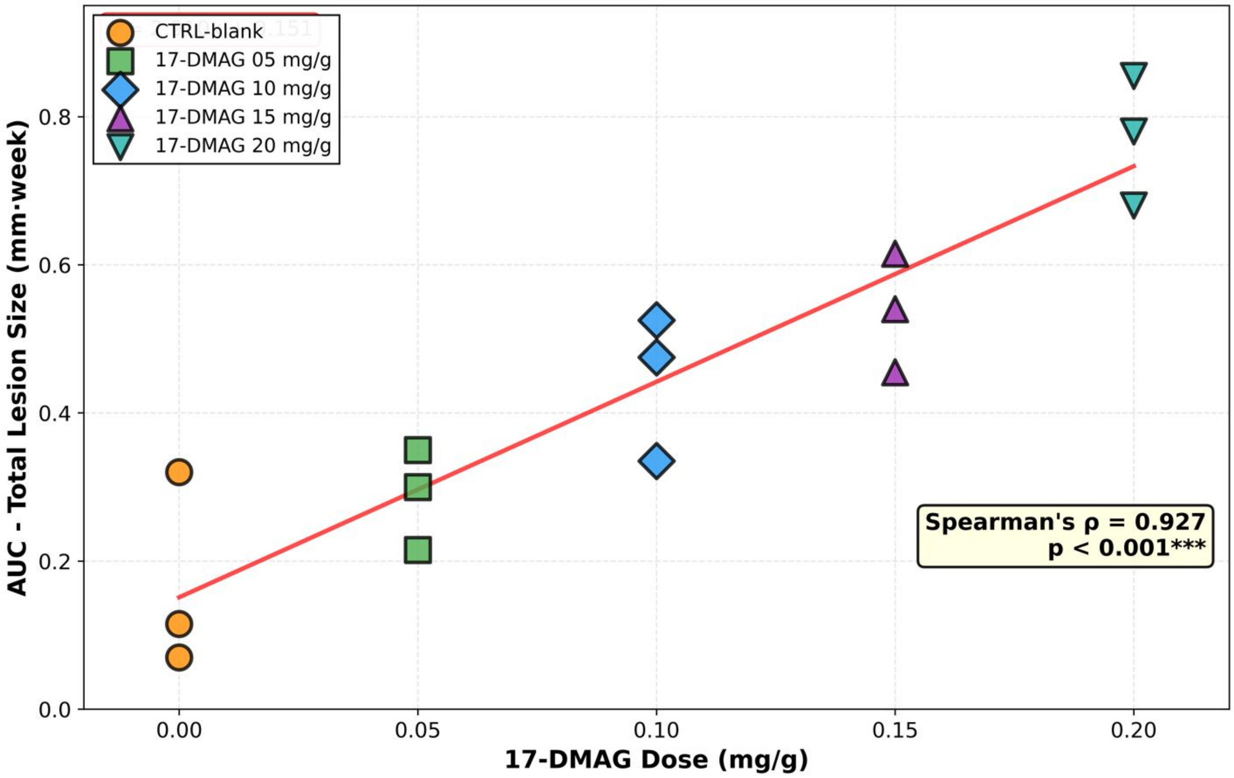

Figure 4S: Dose–response correlation analysis of CMC hydrogels containing 17-DMAG at different concentrations with lesion size. Spearman rank correlation analysis was performed to assess the relationship between 17-DMAG concentration in CMC and cumulative dermal toxicity (expressed as area under the curve, AUC) in uninfected BALB/c mice over a 4-week treatment period. Each data point represents an individual animal treated with either the vehicle control (CTRL-blank) or CMC hydrogel containing 0.05, 0.10, 0.15, or 0.20 mg/g of 17-DMAG. The x-axis shows the 17-DMAG concentration, while the y-axis displays the cumulative lesion burden calculated as AUC. Data points are color-coded by group: orange circles (CTRL-blank), green squares (0.05 mg/g), blue diamonds (0.10 mg/g), purple triangles (0.15 mg/g), and inverted purple triangles (0.20 mg/g). The red solid line represents the linear regression fit, with the equation $y = 2.910x + 0.151$. Statistical significance is indicated by Spearman's rank correlation coefficient ($\rho = 0.927$, $p < 0.001$).

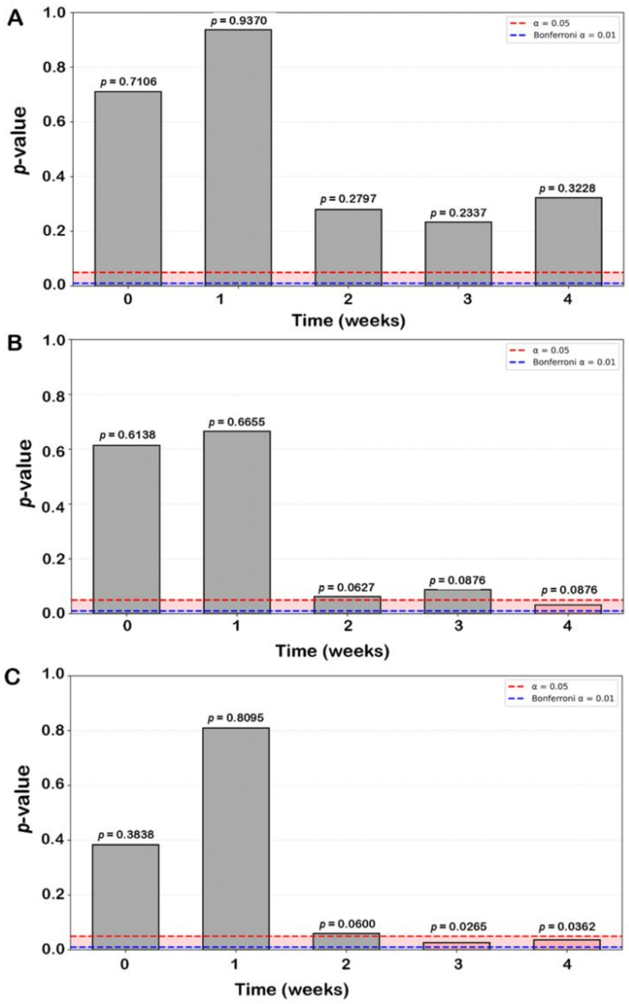

Figure 5S: Pairwise independent *t*-test analyses comparing 17-DMAG treatments to the untreated control at each time point. This figure presents the *p*-value distributions across the 4-week treatment period for CMC hydrogels containing 0.05, 0.10, 0.15, or 0.20 mg/g of 17-DMAG compared with untreated controls. The red and blue dashed lines indicate Bonferroni-corrected significance thresholds at $\alpha = 0.05$ and $\alpha = 0.01$, respectively. (A) Independent *t*-test comparisons of lesion sizes in mice treated with CMC hydrogel containing 0.05 mg/g 17-DMAG *versus* untreated controls across all time points. No statistically significant differences were observed at any time point (week 0: $p = 0.7106$; week 1: $p = 0.9370$; week 2: $p = 0.2797$; week 3: $p = 0.2337$; week 4: $p = 0.3228$). (B) Independent *t*-test comparisons of lesion sizes in mice treated with CMC hydrogel containing 0.10 mg/g 17-DMAG *vs.* untreated controls. No statistically significant differences were detected through week 2 (week 0: $p = 0.6138$; week 1: $p = 0.6655$; At weeks 2 and 3, the *p*-value approached significance ($p = 0.0627$ and $p = 0.0876$, respectively), and by Week 4, the difference reached statistical significance at the $\alpha = 0.05$ level ($p = 0.0319$). (C) Independent *t*-test comparisons of lesion sizes in mice treated with CMC hydrogel containing 0.15 mg/g 17-DMAG *vs.* untreated controls. Between weeks 0 and 2, no significant differences were observed (week 0: $p = 0.3838$; week 1: $p = 0.8095$; week 2: $p = 0.0600$). At week 3, the strongest effect was detected ($p = 0.0265$), and by week 4, the treatment again reached statistical significance ($p = 0.0362$) at the $\alpha = 0.05$ level.

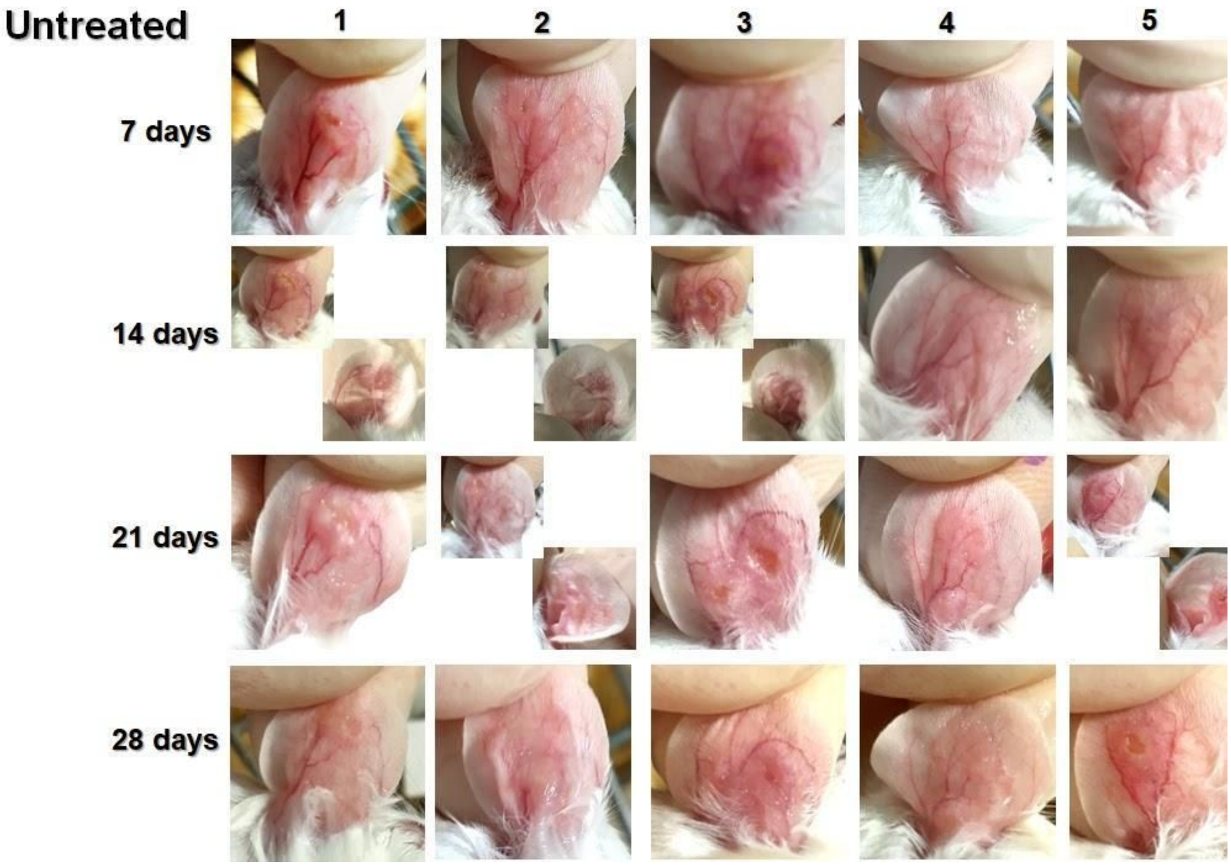

Untreated
1
2
3
4
5
7 days
14 days
21 days
28 days

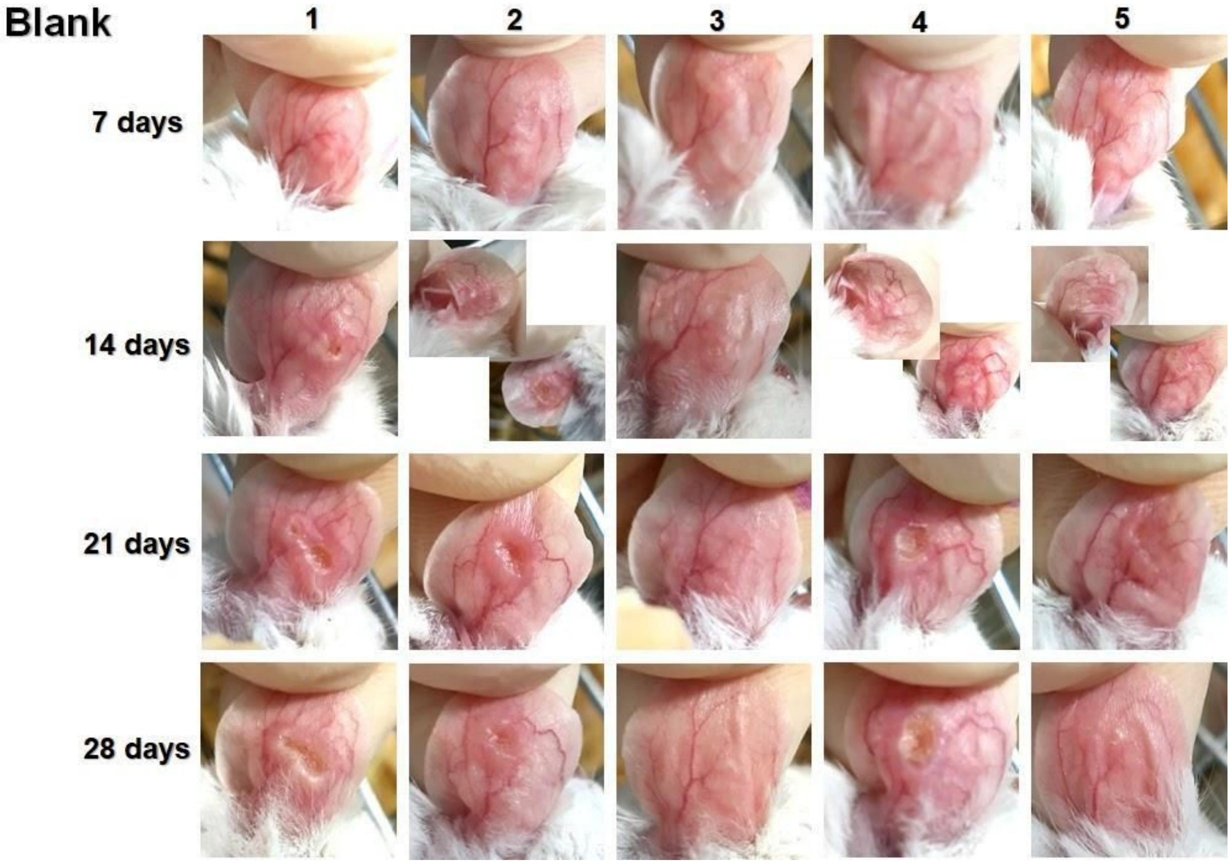
Blank
1
2
3
4
5
7 days
14 days
21 days
28 days

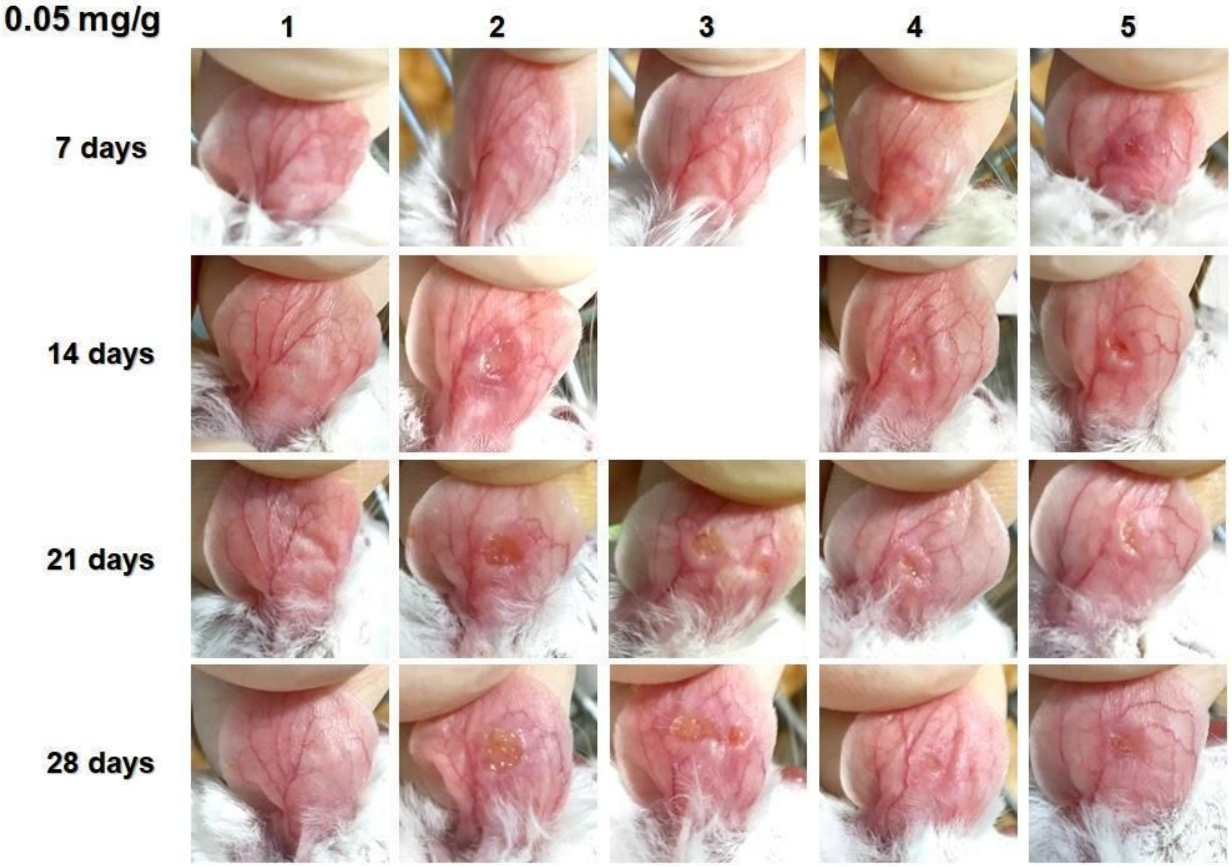
0.05 mg/g
1
2
3
4
5
7 days
14 days
21 days
28 days

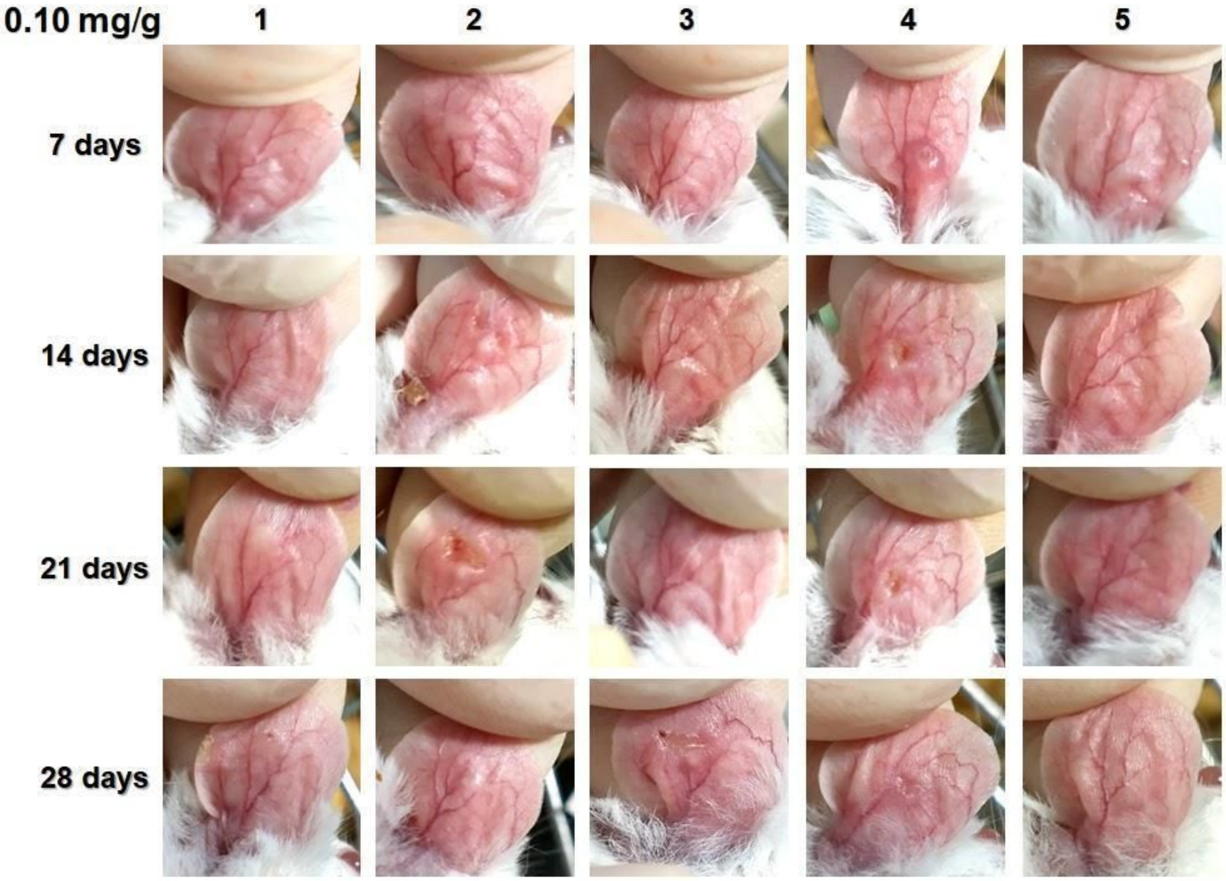
0.10 mg/g
1
2
3
4
5
7 days
14 days
21 days
28 days

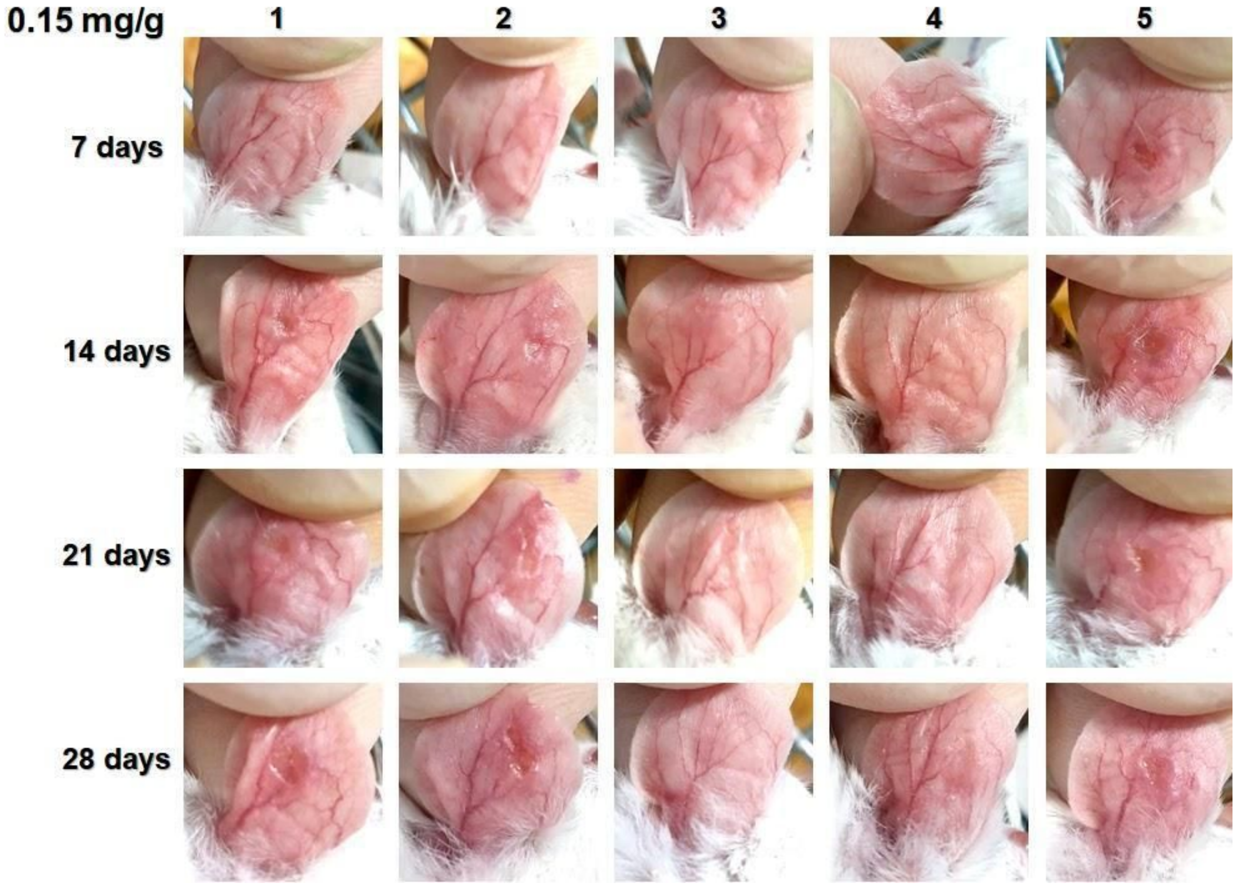

Figure 6S: Macroscopic appearance of ear lesion progression in BALB/c mice infected with *L. braziliensis*. Pannels correspond to images obtained from each animal topically treated with CMC hydrogels containing 0.05, 0.10, 0.15 mg/g of 17-DMAG for four weeks (28 days), as well as control groups, untreated and blank treated with 17-DMAG-free CMC hydrogel. Images were acquired each seven days until 28 days of follow-up.
